# Global, regional, and national burden of injury associated internal hemorrhage in the abdomen and pelvis from 1990-2021

**DOI:** 10.1038/s43856-026-01560-3

**Published:** 2026-04-13

**Authors:** Xiaochen Feng, Xiuli Zhu, Minghao Zou, Wenxuan Zhou, Qianjia Liu, Yan Lu, Wuyu Chen, Queran Lin, Fuchen Liu, Susu Luo, Hui Liu

**Affiliations:** 1 The Third Department of Hepatic Surgery, Eastern Hepatobiliary Surgery Hospital, Shanghai, China; 2 Department of Gastroenterology, Division of Life Sciences and Medicine, The First Affiliated Hospital of USTC, University of Science and Technology of China, Hefei, China; 3 Department of Primary Care and Public Health, School of Public Health, Faculty of Medicine, WHO Collaborating Centre for Public Health Education and Training, Imperial College London, London, UK; 4 Clinical Research Design Division, Guangdong Provincial Key Laboratory of Malignant Tumor Epigenetics and Gene Regulation, Breast Tumor Center, Clinical Research Center, Sun Yat-Sen Memorial Hospital, Sun Yat-Sen University, Guangzhou, China; 5 Moores Cancer Center, University of California San Diego, San Diego, CA USA

**Keywords:** Epidemiology, Public health

## Abstract

**Background:**

Injury associated internal hemorrhage in the abdomen and pelvis (IAIHAP) is a major cause of preventable deaths, particularly in conflict zones. However, its global burden remains inadequately investigated. This study aimed to assess the burden of IAIHAP from 1990 to 2021.

**Method:**

Using data from Global Burden of Disease Study 2021, this study analyzed IAIHAP burden across 204 countries and territories. Incidence, prevalence, and Years Lived with Disability (YLDs) were estimated. Temporal trends were analyzed using join-point regression. Burden estimates were stratified by sex, age, region, and socio-demographic index (SDI).

**Results:**

Despite a significant decline in global age-standardized-incidence-rate (ASIR) from 186.44 to 135.40 per 100,000 population between 1990 and 2021 [Average Annual Percent Change (AAPC): −1.74], IAIHAP remains a substantial health challenge in 2021 with 10.59 million incident cases and 0.69 million YLDs. The decline is more pronounced in males (AAPC: −2.58) compared to females (AAPC: −0.92). High-middle SDI regions demonstrate the largest decline (AAPC: −2.35), while low SDI regions show modest decreases (AAPC: −0.72) with greater fluctuation. Conflict-affected countries, predominantly in low SDI regions, experience significant increases, with Afghanistan showing the highest rise (AAPC: 8.31).

**Conclusions:**

While global IAIHAP burden has declined over the past three decades, significant disparities persist across regions and development levels. The burden remains particularly high in conflict-affected and low-resource settings, highlighting the need for targeted interventions and improved emergency care infrastructure in vulnerable regions.

**Clinical trial number:**

Not applicable

## Plain language summary

Internal bleeding in the abdomen and pelvis is a dangerous injury that can quickly become life-threatening. We studied how often these injuries occur worldwide and how they have changed from 1990 to 2021. Using global health data from 204 countries, we looked at the number of new cases, long-term disability, and trends over time. We found that although the overall global rate of these injuries has dropped, millions of people are still affected each year. The burden remains especially high in countries with limited resources or ongoing conflict, such as Afghanistan and Yemen. These results show that improving emergency care and strengthening health systems, especially in vulnerable regions, is essential to reduce preventable deaths in the future.

## Abstract (web summary)

Feng et al. analyze the global burden of abdominal and pelvic injury-related hemorrhage across 204 countries from 1990 to 2021. They find that despite an overall decline, substantial disparities linked to development level, sex, and conflict exposure persist, with the burden remaining high in vulnerable settings.

## Introduction

Uncontrollable hemorrhage is a major cause of injury related deaths, accounting for more than 80% of military fatalities.^1^ Beyond military conflicts, severe hemorrhage is responsible for over 35% of pre-hospital deaths and nearly 40% of deaths within the first 24 h following injury.^2^ Injury associated internal hemorrhage in abdomen and pelvis (IAIHAP) refers to internal bleeding resulting from vascular rupture of abdominal tissues, organs, or pelvis due to severe trauma. Notably, injury-associated hemorrhage is a major contributor to “preventable deaths” (potentially survivable patients who ultimately died), with over two-thirds of lethal bleeding occurring in the trunk,^3^ and 64% of such truncal injuries specifically located in the abdomen and pelvis.^4^

Blunt trauma to abdominal organs represents the predominant mechanism leading to internal bleeding in the region. The spleen and liver are particularly susceptible to hemorrhage in blunt injuries, contributing significantly to organ injury-related mortality.^5^ The pelvis, located at the inferior aspect of the trunk, comprises the pelvic girdle and perineum, housing urinary and reproductive organs.^6^ This anatomically complex region is highly vulnerable to internal hemorrhage following trauma. Severe pelvic fractures, often termed “killing fractures” due to their refractory hemorrhage, epitomize the lethal risk of major IAIHAP.^7–10^

Pelvic and abdominal injuries are closely linked, as pelvic fractures frequently coexist with abdominal organ damage and their occurrence increases with trauma severity^11,12^ Age-related differences exist, with adults showing greater susceptibility to pelvic injuries, whereas children more often sustain concurrent splenic or hepatic injuries.^13^ In recent years, the global burden of injuries and health crises induced by armed conflicts and terrorism has been exacerbated significantly. Taking the ongoing Russia-Ukraine war as an example, it has resulted in over 70,000 Ukrainian military fatalities and more than 40,000 civilian deaths or injuries,^14,15^ collapsing healthcare infrastructure and amplifying risks of infectious disease outbreaks and mental health disorders.^16,17^ Preventable deaths in these settings are often driven by delayed evacuation, suboptimal hemorrhage control, and limited access to blood products.^18,19^ Advances in prehospital management, including permissive hypotension, hemostatic agents, and prehospital plasma, have shown significant reductions in mortality from abdominopelvic hemorrhage.^20–22^ Integrating these evidence-based interventions into trauma systems should be a global priority to mitigate preventable deaths, particularly in regions with high injury burdens.

Previous analyzes using the Global Burden of Diseases, Injuries, and Risk Factors Study (GBD) have primarily focused on injury causes, such as falls, drowning, transport injuries.^23–26^ However, few articles have examined the nature of injury, which often provides more relevant insights into injury consequences than the precipitating cause.^27^ IAIHAP represents a particularly significant nature-of- injury category, especially in battlefield scenarios.^1–4^

This study provides a comprehensive analysis of the global, regional, and national burden of IAIHAP from 1990 to 2021. The findings show that while the global burden of IAIHAP has significantly declined over this period, substantial disparities persist. The burden remains relatively high in conflict-affected areas and low-resource settings. These findings underscore that IAIHAP continues to be a major public health challenge linked to global inequality, highlighting the urgent need for targeted interventions and strengthened trauma care infrastructure in vulnerable regions.

## Methods

### Data sources and study design

This study utilized a retrospective trend design to analyze data on incidence, prevalence, and Years Lived with Disability (YLDs), along with corresponding 95% uncertainty intervals (UIs), derived from the GBD 2021, which provides comprehensive estimates of disease burden across 371 diseases and injuries, including 40 injury categories.^27^ The study encompassed 204 countries and territories, with 21 countries having subnational locations, spanning from 1990 to 2021 (https://vizhub.healthdata.org/gbd-results/). Data were stratified by sex and 25 age groups. Estimates for the nature of injury (e.g., internal hemorrhage) were not generated directly by the Disease Modeling-Meta Regression (DisMod-MR) models. Instead, they were derived through a multi-step process that translates cause-specific incidence into cause-nature pairs. This process relied primarily on two components: cause-nature matrices and probabilities of permanent health loss. The cause-nature matrices were constructed using dual-coded (both external cause and nature of injury) hospital and emergency department datasets from numerous countries worldwide. These matrices define the proportion of each cause of injury (e.g., falls) that results in a specific nature of injury (e.g., internal hemorrhage in abdomen and pelvis). The proportions were estimated simultaneously using Dirichlet models, which ensure all proportions sum to 1 and allow for borrowing information across age, sex, care setting (inpatient/outpatient), and country income level. Subsequently, these matrices were applied to the cause-specific incidence estimates from DisMod-MR to generate incidence for each cause-nature pair.

To handle cases with multiple injuries, a nature-of-injury severity hierarchy, established based on long-term disability weights derived from pooled follow-up studies, was applied to assign the single most severe nature of injury to each case. Finally, the probability of developing permanent health loss for each nature-of-injury, stratified by age and care setting, was estimated using longitudinal data from follow-up studies and the Medical Expenditure Panel Survey (MEPS) via a logit-linear mixed-effects regression model. This probability was used to distinguish between short-term and long-term prevalence and YLDs.^27,28^ Beyond administrative and clinical data, the estimation incorporated population-representative surveys, such as the World Health Survey (WHS) and MEPS to capture injuries outside formal healthcare settings and for analyzing long-term outcomes. Furthermore, supplementary sources like collaborator accounts and news reports were used to ensure comprehensive coverage of episodic events, such as conflicts and disasters.^27^

To estimate non-fatal injury incidence from fatal discontinuities (e.g., conflict, terrorism, disasters), for which direct incidence data are unavailable, this study applied incidence-to-mortality ratios to mortality estimates. The mortality inputs for this process were fundamentally derived from databases including the Armed Conflict Location and Event Dataset, the Global Terrorism Database,^29^ Uppsala Conflict Data Program,^30^ the International Institute for Strategic Studies,^31^ the Social Conflict Analysis Database and vital registration systems.^32^ Disaster data were obtained from International Disaster Database, maintained by the Center for Research on the Epidemiology of Disasters,^33^ supplemented by news reports.^27^ The current study was conducted methodically, following the Guidelines for Accurate and Transparent Health Estimates Reporting (GATHER).^34^

### Case definition, measures, and geographic hierarchy

This study focused on internal hemorrhage in the abdomen and pelvis, categorized under “injuries by nature” in the GBD estimates. To estimate the incidence and burden of this specific nature of injury, the GBD study employs cause-nature matrices. These matrices, derived from dual-coded datasets where both the external cause (e.g., fall) and the specific nature of injury (e.g., internal hemorrhage) are recorded, provide the probability distribution of various natures of injury for each cause. This study then examined all categories under the “injuries” section to identify all potential causes of internal hemorrhage in these regions. In 2021, injuries were classified as level one causes, while specific subcategories were classified as level two and level three causes. Level two causes included unintentional injuries, transport injuries, self-harm and interpersonal violence. At the level three subcategories, cause included road injuries, other transport injuries, falls, drowning, fire, heat, and hot substances, poisonings, exposure to mechanical forces, adverse effects of medical treatment, animal contact, foreign body, exposure to forces of nature, environmental heat and cold exposure, self-harm, interpersonal violence, police conflict and executions, conflict and terrorism. ICD codes corresponding to the causes of injury involved in this study are presented in the supplementary materials (Supplementary Data 1). Consequently, by applying these cause-nature matrices, this study estimated the burden of IAIHAP attributable to each of these underlying causes of injury.

The study examined three primary outcome measures: incidence, prevalence, and YLDs. Incidence captured new cases of IAIHAP, while prevalence represented the total number of cases at a given time point. YLDs quantified the burden of health loss due to disability. Age-standardized rates (ASRs), including Age-Standardized Incidence Rate (ASIR), Age-Standardized Prevalence Rate (ASPR), Age-Standardized YLDs Rate (ASYR), were calculated for comparison across populations.

The study employed a hierarchical geographic structure, including global, 7 super-regions, 21 regions, and 205 countries levels (Supplementary Data 2). Locations were classified into five Socio-demographic Index (SDI) quintiles, which reflect development status based on per capita income, educational attainment, and fertility rates under age 25.^27^ The SDI values ranged from 0 to 1, with countries grouped into high, high-middle, middle, low-middle, and low quintiles (Supplementary Data 3).

### Statistics analysis

#### Disease burden estimation

The study followed the overarching methodology of GBD 2021 for estimating disease burden.^35^ Input data for IAIHAP were drawn from surveillance systems, registries, and published studies, and modeled using DisMod-MR version 2.1, a Bayesian meta-regression tool widely employed in GBD epidemiological modeling. After data adjustment, DisMod-MR2.1 was used to estimate prevalence and incidence rates. For injuries with significant fatal and non-fatal inconsistencies, an excess mortality rate model was employed using the meta-regression-Bayesian, regularized, trimmed (MR-BRT) tool, with age, sex, and the Healthcare Access and Quality (HAQ) Index as predictors.

For fatal discontinuities, including state actor violence, exposure to forces of nature, or conflict and terrorism, an alternative to the standard DisMod-MR 2.1 approach was applied. In these cases, incidence was calculated using incidence-to-mortality ratios. The study prioritized incidence measures over prevalence for analyzing acute events like IAIHAP, as incidence better captures the immediate impact of injury.^36^ While prevalence data were collected and presented, the primary results and discussion focused on incidence and YLDs, with YLDs being calculated based on prevalence to assess health loss due to disability.^27^ The cause proportion of IAIHAP was calculated by dividing the cause-specific ASR by the overall ASR for all injuries combined. These calculations were based on the GBD 2021 point estimates to evaluate the relative contribution of each injury cause to the overall burden.

#### Trend analysis

Temporal trends were analyzed using the average annual percentage change (AAPC) from Joinpoint regression. This approach provided a robust and inferential summary of the entire trend period, mitigating the effect of year-to-year noise and endpoint volatility through model-based estimation and confidence intervals. The result is a standardized metric that facilitates the comparison of long-term trends across different populations and regions. The calculation of AAPC was performed as follows.

The Annual percentage change (APC) was calculated for each join-point, using the formula:

$${{APCi}}=(exp ({{bi}})-1)\times 100.$$

The Average Annual Percent Change (AAPC), along with 95% confidence intervals (CIs) was computed as the weighted mean of individual APCs to reflect the overall trend:

$${AAPC}=(exp \left(\frac{\Sigma {wibi}}{\Sigma {wi}}\right)-1)\times 100,$$

where *b*_*i*_ is the slope coefficient for the *i*th segment with *i* indexing the segments in the desired range of years, and *w*_*i*_ is the length of each segment in the range of years.^37^

Denote the normalized weight as:

$${\widetilde{w}}_{i}={w}_{i}/\sum {w}_{j}$$

AAPC can be rewritten as:

$${AAPC}=\left(exp \left(\sum {\widetilde{w}}_{i}{b}_{i}\right)-1\right)\times 100$$

An approximate 100 (1−*α*)% CIs for true AAPC is

$$\left({{{\rm{AAPC}}}}_{{{\rm{L}}}({{\rm{\alpha }}})},\,{{{\rm{AAPC}}}}_{{{\rm{U}}}({{\rm{\alpha }}})}\right)$$, where

$${{AAPC}}_{L(\alpha )}=\left\{\exp \left[\log \left(\left({AAPC}/100\right)+1\right)-{z}_{1-\alpha /2}\sqrt{\sum {\widetilde{w}}_{i}^{2}{\hat{\sigma }}_{i}^{2}}\right]-1\right\}\times 100$$

$${{AAPC}}_{U(\alpha )}=\left\{\exp \left[\log \left(\left({AAPC}/100\right)+1\right)+{z}_{1-\alpha /2}\sqrt{\sum {\widetilde{w}}_{i}^{2}{\hat{\sigma }}_{i}^{2}}\right]-1\right\}\times 100$$

are the lower and upper confidence limits of the interval, **Ζ**_**α**_ is the **α**^**th**^ quantile of the standard normal distribution, and ${\hat{{{\boldsymbol{\sigma }}}}}_{{{\bf{i}}}}^{{{\bf{2}}}}$ denotes the estimated variance of ***b***_***i***_ obtained from the fit of the join-point model.

Trends were classified as upward if both the AAPC estimate and the lower 95% CI exceeded 0, downward if both the AAPC estimate and the upper bound were below 0, and flat if the 95% CI included 0.^38^

#### Software and tools

This study employed smoothing spline regression to model the association between IAIHAP disease burden and SDI across 204 countries and territories. Locally Weighted Scatterplot Smoothing method was used to fit smooth splines, determining the degree, number, and location of nodes on the basis of the data and the span parameter. Spearman correlation analysis determined the association (rho, *P*-value) between the ASIR and SDI, with *P* < 0.05 indicating statistical significance.

Statistical analyses were performed using a combination of software packages: R (version 4.4.0) for statistical analysis, the Join-point Regression Program for trend analysis, and GraphPad Prism 9.0 (GraphPad Software, Inc) for visualization.

#### Ethics approval and consent to participate

The GBD Study itself adheres to strict ethical protocols. The collection and processing of data within the GBD framework were approved by the Institutional Review Board (IRB) of the University of Washington, which waived the need for individual informed consent for the aggregated, de-identified data used in GBD analyses. Further details regarding GBD’s ethical standards and data governance can be found on the official GBD website.

## Results

### Global burden and temporal trends of IAIHAP (1990–2021)

Between 1990 and 2021, the global burden of IAIHAP demonstrated consistent decreasing trends, as shown in Supplementary Data 4 and 5. The ASIR showed a significant decline (AAPC: −1.74, 95% CI: −1.85 to −1.62), from 186.44 to 135.40 per 100,000 population. Similarly, the ASPR decreased (AAPC: −0.97, 95% CI: −0.98 to −0.95) from 93.76 to 63.84 per 100,000 population. The age-standardized YLD rate also exhibited a modest reduction (AAPC: −0.12, 95% CI: −0.13 to −0.12) from 12.19 to 8.38 per 100,000 population over the study period. Despite these declining trends, IAIHAP remained a substantial global health challenge in 2021, with the global incidence reaching 10.59 million cases (95% UI: 8.73–12.67), prevalence at 5.35 million cases (5.03–5.70), and YLDs amounting to 0.69 million (0.48–0.93).

**Fig. 1:**
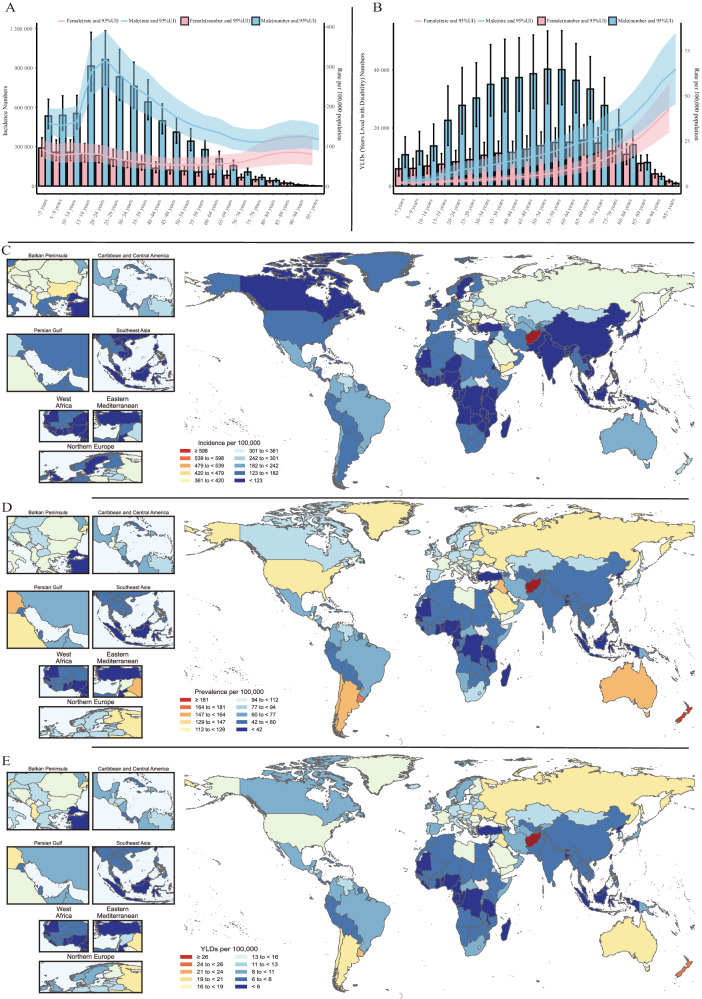
Disease burden of injury associated internal hemorrhage in abdomen and pelvis (IAIHAP) in 2021. **A** Age-specific incidence numbers and rates of IAIHAP by sex, 2021. Data points and lines represent the mean estimates, error bars and bands represent the 95% uncertainty interval. **B** Age-specific YLDs numbers and rates of IAIHAP by sex, 2021. Data points and lines represent the mean estimates, error bars and bands represent the 95% uncertainty interval. **C** Age-standardized incidence rate (ASIR) across 204 countries. Map data from Natural Earth, public domain. **D** Age-standardized prevalence rate (ASPR) across 204 countries. Map data from Natural Earth, public domain. **E** Age-standardized years lived with disability (YLDs) rate (ASYR) across 204 countries. Map data from Natural Earth, public domain.

These overall trends masked important sex-specific patterns. Sex-stratified analysis revealed disparate trends, with males showing a more pronounced decline in ASIR (AAPC: −2.58, 95% CI: −2.69 to −2.46; from 275.84 to 199.32 per 100,000) compared to females (AAPC: −0.92, −1.11 to −0.72; from 95.20 to 70.07 per 100,000). Similar sex-based patterns were observed in prevalence and YLDs (Supplementary Data 4 and 5). Age-specific analysis showed highest incidence in males particularly among those aged 15–29 years (older adolescents and young adults), with a male-to-female ratio of approximately 3:1, followed by gradual declines with age while maintaining male predominance (Fig. 1A). The YLD burden peaked in middle adulthood for both sexes, with less pronounced sex disparities compared to incidence (Fig. 1B).

Geographically, IAIHAP burden demonstrated distinct regional patterns in 2021. Central Europe reported the highest ASIR of 333.80 per 100,000 population (95% UI: 249.63–431.37), followed by Eastern Europe (327.49 per 100,000 population, 258.45–401.60), while significantly lower ASIRs were observed in East Asia (106.82 per 100,000 population, 84.94–133.23) and Southeast Asia (109.78 per 100,000 population, 90.08–131.22) (Supplementary Data 4). At the national level, the highest ASIRs were observed in Afghanistan, Albania, Slovenia, Yemen, and Bulgaria (Fig. 1C, and Supplementary Data 6). The distribution of prevalence and disability burden showed different patterns from incidence. While Latin America and most of Oceania showed lower incidence rates, high prevalence rates were observed in Oceania (particularly New Zealand and Australia) and South America (Uruguay and Argentina) (Fig. 1D, and Supplementary Data 6). The YLD burden similarly showed regional variations, with the highest impacts in South Asia (Afghanistan), the Middle East (Iraq), Oceania (Australia), and South America (Uruguay) (Fig. 1E, and Supplementary Data 6).

**Fig. 2:**
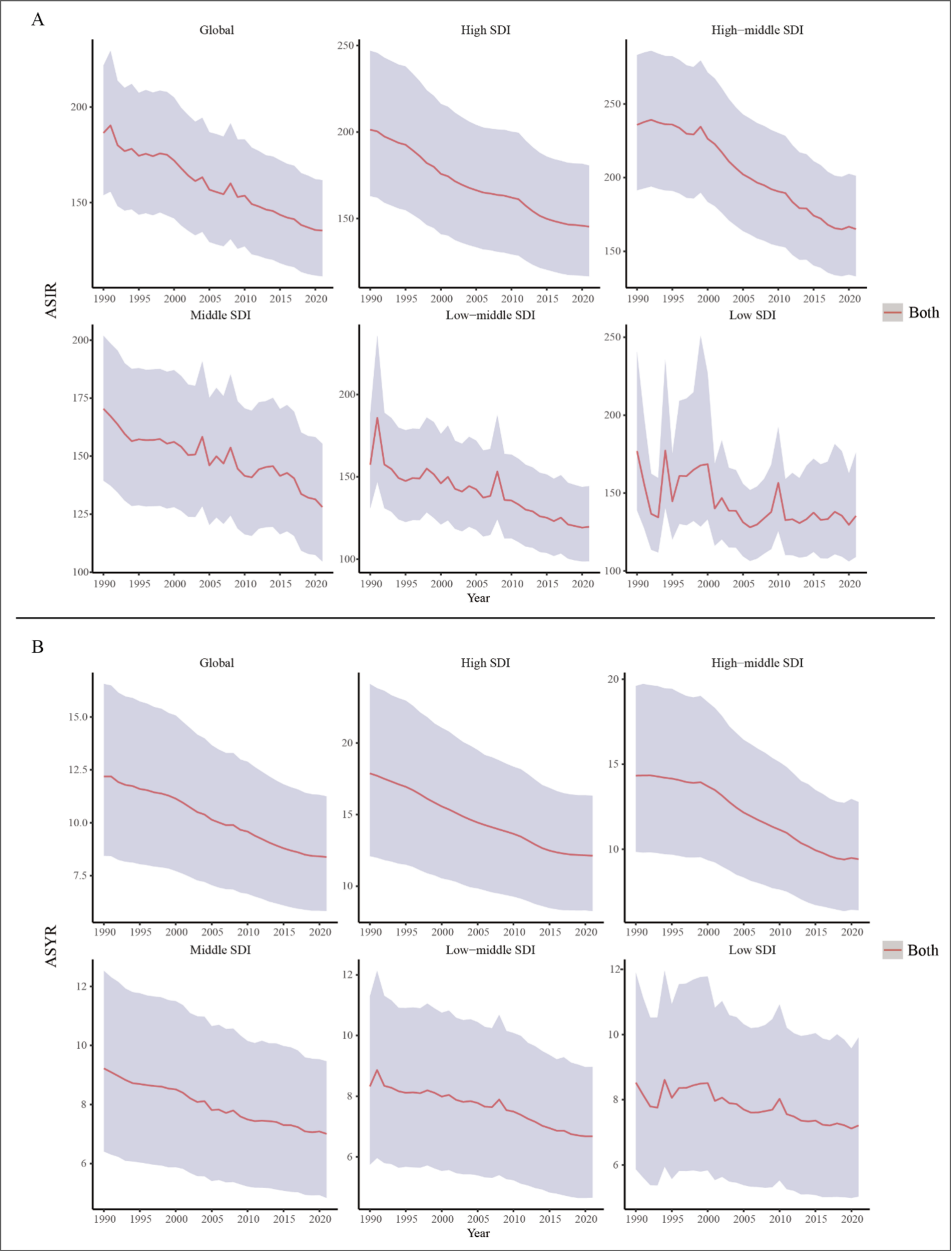
Temporal trends of injury associated internal hemorrhage in abdomen and pelvis (IAIHAP) across five sociodemographic index (SDI) quintiles, 1990–2021. **A** Temporal trends in age-standardized incidence rate (ASIR) of IAIHAP by SDI quintile, 1990–2021. The lines represent the mean estimates, error bands represent the 95% uncertainty interval. **B** Temporal trends in age-standardized years lived with disability (YLDs) rate (ASYR) of IAIHAP by SDI quintile, 1990–2021. The lines represent the mean estimates, error bands represent the 95% uncertainty interval.

SDI regional analysis revealed heterogeneous patterns in IAIHAP burden from 1990 to 2021. High-middle SDI regions, with the highest initial ASIR of 235.81 per 100,000 population in 1990, demonstrated the most substantial reduction (AAPC: −2.35, 95% CI: −2.47 to −2.23), declining to 165.09 per 100,000 population by 2021. High SDI regions followed with the second largest decline (AAPC: −1.88, 95% CI: −1.93 to −1.83), with ASIR reducing from 201.35 to 145.38 per 100,000 population. Middle SDI, low-middle SDI, and low SDI regions experienced more modest decreases, with AAPCs of −1.38 (95% CI: −1.59 to −1.18), −1.46 (−1.84 to −1.08), and −0.72 (−1.26 to −0.18), respectively (Supplementary Data 4 and 5). Temporal trends varied, with high-middle and high SDI regions showing relatively smooth, consistent decreases throughout the study period, while low and low-middle SDI regions displayed more volatile trends with notable fluctuation, particularly in the early 1990s and mid and late-2000s (Fig. 2A). Similar patterns were observed in age-standardized YLD rates, though with less fluctuation across all temporal lines (Fig. 2B).

Despite the overall regional declining trends, several countries experienced significant increases in IAIHAP burden. Afghanistan experienced the most dramatic increase (AAPC: 8.31, 95% CI: 4.27 to 12.34), with ASIR rising to 657.45 per 100,000 population in 2021 (Table 1, and Supplementary Data 5 and 6). The trend in Afghanistan showed two distinct periods of increase: 1990–1998 and 2003–2019, coinciding with periods of political instability (Fig. S1A). Yemen experienced the second-highest increase (AAPC: 6.60, 95% CI: 5.46 to 7.73), with a particularly sharp rise between 2014 and 2018 (Fig. S1B). Similarly, Lesotho (AAPC: 1.68, 1.48 to 1.89) experienced increases during 1990–1996, 1996–1998, and 1998–2009 (Fig. S1J). Other conflict-affected countries, including Rwanda, Libya, Burundi, South Sudan, Congo, Armenia, and Mali, also showed notable but statistically insignificant increases (Figs. S1C–S1I, and Table 1).

**Table 1:** The top ten countries with AAPC of ASIR and corresponding wars/conflicts

Location	AAPC of ASIR, 1990–2021	Wars or conflicts
Afghanistan (1st)	8.31 (4.27, 12.34)	Afghanistan Civil War, 1992–1996; Afghanistan War, 2001–2021
Yemen (2nd)	6.60 (5.46, 7.73)	The First Yemen Civil War, 1994; The Second Yemen Civil War, 2014 to present
Rwanda (3rd)	4.56 (−21.01, 30.13)	Rwanda Civil War, 1990–1994
Libya (4th)	3.92 (−0.38, 8.23)	The First Libya Civil War, 2011; The Second Libya Civil War, 2014–2020
Burundi (5th)	3.89 (−0.45, 8.24)	The Burundi Civil War, 1993–2005
South Sudan (6th)	3.26 (−0.10, 6.63)	The Second Sudan Civil War, 1983–2005; The South Sudan Civil War, 2013 to present
Congo (7th)	3.21 (−2.86, 9.28)	The First Congo War, 1996–1997; The Second Congo War, 1998–2003
Armenia (8th)	2.89 (−3.24, 9.02)	The Nagorno-Karabakh Conflict, 1988–1994; The Nagorno-Karabakh Conflict, 2020 to present
Mali (9th)	1.80 (−0.80, 4.40)	The Mali Civil War, 1990–1996; The Conflict in Northern Mali, 2012; The Mali Serval Operation 2013–2014
Lesotho (10th)	1.68 (1.48, 1.89)	Military coup, 1991, 1994, 2014

*AAPC* average annual percent change, *ASIR* age-standardized-incidence-rate.

Although ASYR increases were modest compared to ASIR, they highlight a significant long-term disability burden. These changes are crucial, as they indicate persistent disability impacts even in regions where acute injury rates show minimal changes. For example, while Syria ranked 19th in ASIR magnitude with a non-significant increase (AAPC: 0.6, 95% CI: −2.01 to 6.55), it showed a significant ASYR rise (AAPC: 0.20, 0.11 to 0.28), ranking second overall, with a sharp peak in 2015 (Fig. S2B). Significant ASYR increases were also observed in Libya (AAPC: 0.19, 95% CI: 0.10 to 0.28), Burundi (AAPC: 0.16, 0.05 to 0.27), Yemen (AAPC: 0.15, 0.13 to 0.18), Lesotho (AAPC: 0.12, 0.12 to 0.13), South Sudan (AAPC: 0.09, 0.03 to 0.15), Papua New Guinea (AAPC: 0.07, 0.03 to 0.11), Belize (AAPC: 0.07, 0.06 to 0.09), and Botswana (AAPC: 0.06, 0.05 to 0.07) (Fig. S2, and Table 2)

**Table 2:** The top ten countries with AAPC of ASYR and corresponding wars/conflicts

Location	AAPC of ASYR, 1990–2021	Wars or conflicts
Rwanda (1st)	0.29 (−0.11, 0.70)	Rwanda Civil War, 1990–1994
Syria (2nd)	0.20 (0.11, 0.28)	The Syrian Civil War, 2011 to present
Libya (3rd)	0.19 (0.10, 0.28)	The First Libya Civil War, 2011; The Second Libya Civil War, 2014–2020
Burundi (4th)	0.16 (0.05, 0.27)	The Burundi Civil War, 1993–2005
Yemen (5th)	0.15 (0.13, 0.18)	The First Yemen Civil War, 1994; The Second Yemen Civil War, 2014 to present
Lesotho (6th)	0.12 (0.12, 0.13)	Military coup, 1991, 1994, 2014
South Sudan (7th)	0.09 (0.03, 0.15)	The Second Sudan Civil War, 1983–2005; The South Sudan Civil War, 2013 to present
Papua New Guinea (8th)	0.07 (0.03, 0.11)	Bougainville Civil War, 1988–1998
Belize (8th)	0.07 (0.06, 0.09)	Belize-Guatemala conflict
Afghanistan (10th)	0.06 (−0.02, 0.13)	Afghanistan Civil War, 1992–1996; Afghanistan War, 2001–2021
Botswana (10th)	0.06 (0.05, 0.07)	–

*AAPC* average annual percent change, *ASYR* age-standardized-YLDs-rate, *YLDs* years lived with disability.

### The cause proportion of IAIHAP

**Fig. 3:**
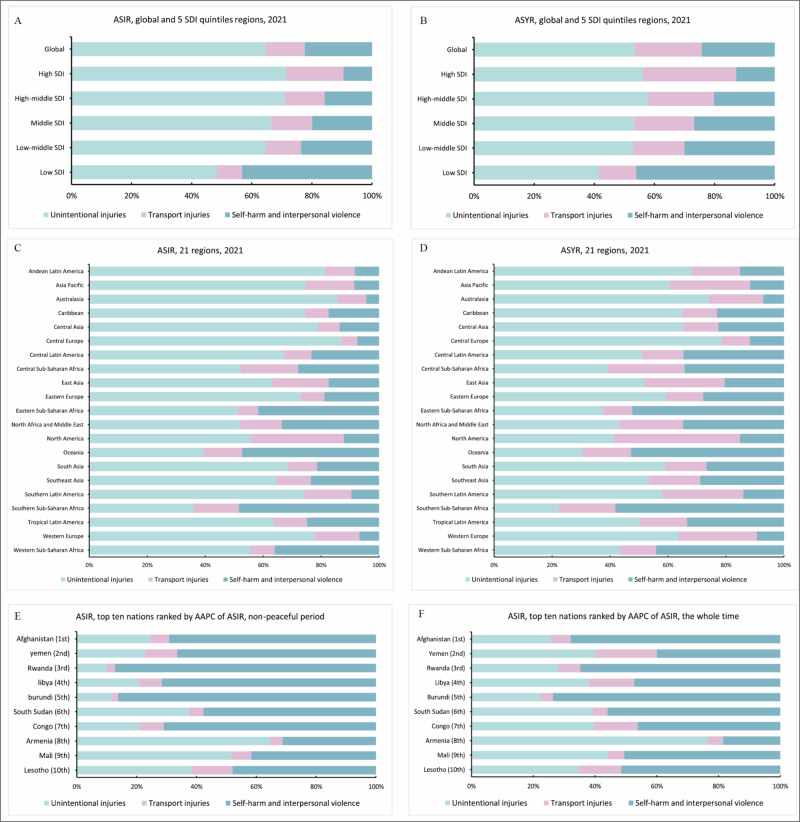
Cause proportion of disease burden for injury associated internal hemorrhage in abdomen and pelvis (IAIHAP) in 2021. **A** Cause proportion of age-standardized incidence rate (ASIR) by five sociodemographic index (SDI) quintiles, 2021; **B** Cause proportion of age-standardized years lived with disability (YLDs) rate (ASYR) by five SDI quintiles, 2021; **C** Cause proportion of ASIR by GBD region, 2021; **D** Cause proportion of ASYR by GBD region, 2021; **E** Cause proportion of top 10 countries/regions with highest AAPC of ASIR, non-peaceful period; **F** Cause proportion of top 10 countries/regions with highest AAPC of ASIR, the whole time, 1990–2021.

In GBD 2021, injuries were hierarchically categorized into three levels: level one (injuries), level two (unintentional injuries, transport injuries, self-harm and interpersonal violence), and level three (specific causes including road injuries, falls, interpersonal violence, conflict and terrorism, etc.). Globally, unintentional injuries dominated the ASIR composition in 2021, accounting for 64.57%, followed by self-harm and interpersonal violence (22.35%) and transport injuries (13.08%). This pattern varies across SDI quintiles, with unintentional injuries remaining predominant but showing a notable trend: the proportion of self-harm and interpersonal violence decreased as SDI level increased (Fig. 3A, and Table S1). At level three, falls were the leading cause in most SDI regions except the low SDI quintile, where interpersonal violence (20.86%) and conflict and terrorism (21.03%) predominated (Table S1). The regional analysis indicated that unintentional injuries were the leading cause in most regions. However, in Oceania, various Sub-Saharan Africa regions, as well as North Africa and the Middle East, self-harm and interpersonal violence were more prevalent (Fig. 3C, and Table S3).

The ASYR composition showed similar but distinct patterns. Globally, unintentional injuries accounted for 53.59%, followed by self-harm and interpersonal violence (24.28%) and transport injuries (22.13%). A clear SDI gradient emerged: higher SDI levels corresponded with increased transport injuries (12.51% to 31.20%) and decreased self-harm and interpersonal violence (46.01% to 12.73%). In the low SDI quintile, self-harm and interpersonal violence were uniquely the predominant cause of ASYR (46.01%) (Fig. 3B, and Table S2). At level three, falls remained dominant globally except in low SDI regions, where interpersonal violence (27.76%) prevailed. Transport injuries, particularly road injuries, showed increasing prominence with higher SDI levels, while interpersonal violence showed the opposite trend (Table S2). Regionally, while unintentional injuries generally predominated, notable exceptions included North America (transport injuries, 43.42%) and Southern Sub-Saharan Africa, Oceania, and Eastern Sub-Saharan Africa (self-harm and interpersonal violence >50%) (Fig. 3D, and Table S4).

Further analysis of cause composition in the top ten countries with highest ASIR-AAPC revealed distinct patterns between peaceful and conflict periods. During non-peaceful periods, self-harm and interpersonal violence dominated, with particularly high proportions in Rwanda (87.21%) and Burundi (86.18%), followed by Libya (71.53%) and Congo (70.95%) (Fig. 3E, and Table S5). In contrast, when examining the entire study period (1990–2021), while self-harm and interpersonal violence remained the leading cause in most countries, their proportions were generally lower, such as in Burundi (73.60%), Afghanistan (67.89%), and Rwanda (64.8%) (Fig. 3F, and Table S6). Notably, Armenia showed a distinct pattern with unintentional injuries as the predominant cause (76.73% for all-time, 64.63% during conflicts), while Yemen and Mali maintained relatively balanced proportions of unintentional injuries and of self-harm and interpersonal violence across both periods. These patterns highlight conflict periods that shift the cause composition toward violence-related injuries.

### COVID-19 impact on IAIHAP burden

GBD 2021 utilized the same primary data input as GBD 2019, with supplemental updates from hospital and emergency department records where more recent site-years were available site-years.^27^ Estimates for the pandemic period (2019–2021) are subject to increased uncertainty, reflecting potential disruptions in global health data reporting systems. Table 3 shows the AAPC of ASIR before and during COVID-19 pandemic. Globally, while the overall declining trend from 1990 to 2021 was evident, the period before COVID-19 (2017–2019) showed a non-significant decrease (AAPC: −1.56, 95% CI: −5.79 to 2.87). During the pandemic period (2019-2021), this trend remained relatively stable (AAPC: −0.57, 95% CI: −3.31 to 2.24). Among SDI regions, only high SDI quintile demonstrated a significant decrease during the pandemic period (AAPC: −0.32, 95% CI: −0.44 to −0.19, *p* = 0.02), while other SDI quintiles showed varying but non-significant changes (Table 3).

**Table 3:** The AAPC of ASIR before and during COVID-19

Location	2017–2019	*P*. Value	2019–2021	*P*. Value
Global	−1.56 (−5.79, 2.87)	0.14	−0.57 (−3.31, 2.24)	0.23
High SDI	−0.42 (−2.12, 1.30)	0.20	−0.32 (−0.44, −0.19)	0.02
High-middle SDI	−0.94 (−4.27, 2.51)	0.18	0.05 (−7.22, 7.90)	0.94
Middle SDI	−3.02 (−15.47, 11.26)	0.22	−1.53 (−8.57, 6.06)	0.23
Low-middle SDI	−2.07 (−10.93, 7.67)	0.22	−0.21 (−4.43, 4.21)	0.65
Low SDI	0.88 (−16.22, 21.46)	0.66	−0.05 (−29.42, 41.53)	0.99

Two-sided *t*-tests are used for statistical test, with no adjustment for multiple comparisons.

*AAPC* average annual percent change, *ASIR* age-standardized-incidence-rate, *SDI* sociodemographic index.

At the national level, trend analyzes based on the modeled estimates indicated shifts for some countries during the pandemic period, although these findings require cautious interpretation due to the aforementioned data limitations. The pandemic period witnessed notable shifts in IAIHAP trends for some countries. Before COVID-19 (2017–2019), 14 countries demonstrated significant changes, predominantly decreases, with Syrian Arab Republic showing the largest decline (AAPC: −26.50, 95% CI: −26.84 to −26.16) and Chad the most substantial increase (AAPC: 5.36, 3.93 to 6.80). During the pandemic (2019–2021), a remarkable shift occurred with six countries or regions showing significant increases in ASIR, such as Guyana (AAPC: 0.63, 95% CI: 0.04 to 1.21), Lithuania (AAPC: 0.59, 0.16 to 1.01), Maldives (AAPC: 0.32, 0.20 to 0.43), Marshall Islands (AAPC: 0.30, 0.22 to 0.38), and Niue (AAPC: 0.24, 0.02 to 0.47) (Supplementary Data 7). These country-specific estimates for the pandemic period are highly model-dependent and should be interpreted with caution and validated using more completed future data.

### The correlation between disease burden and socio-demographic index (SDI)

**Fig. 4:**
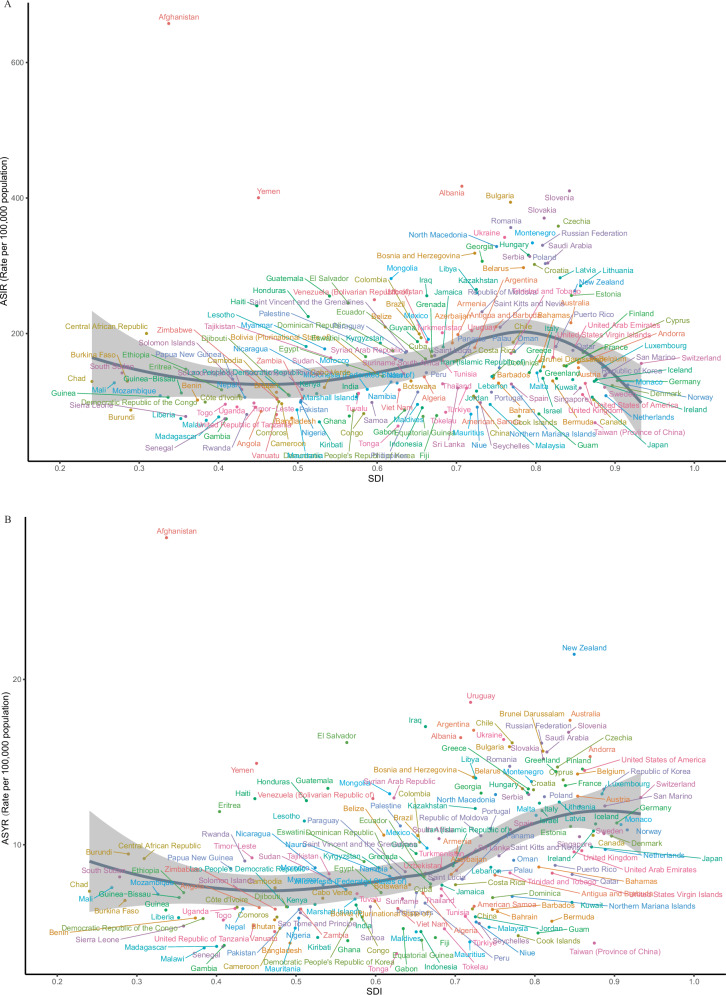
The correlation between Injury associated internal hemorrhage in abdomen and pelvis (IAIHAP) burden and sociodemographic index (SDI) value in 2021. **A** The correlation between age-standardized incidence rate (ASIR) and SDI value in 2021. **B** The correlation between age-standardized years lived with disability (YLDs) rate (ASYR) and SDI value in 2021.

The relationship between IAIHAP burden and societal development showed complex, non-linear patterns in 2021. ASIR across SDI levels showed a moderate positive correlation (Spearman’s rho = 0.35, *p* < 0.01), presenting a U-shaped relationship with higher burdens at both ends of the SDI spectrum (Fig. 4A, and Table S7). Similarly, ASYR showed a stronger positive correlation with SDI (rho =  0.50, *p* < 0.01), though with less pronounced variation at lower SDI levels (Fig. 4B, and Table S7).

Detailed analysis of specific SDI quintiles revealed distinct patterns. In the high SDI quintile, ASIR reported a significant negative correlation (rho = −0.64, *p* < 0.01), suggesting that among developed nations, higher development levels were associated with lower incidence rates (Fig. S3A). Conversely, in the low-middle SDI quintile, both ASIR and ASYR showed positive correlations (ASIR: rho = 0.46, *p* < 0.01; ASYR: rho = 0.52, *p* < 0.01), indicating that within this development range, increasing SDI was associated with higher burden (Fig. S3B, C). No significant correlations were observed in other SDI quintiles, suggesting more complex relationships between development and IAIHAP burden in these SDI quintiles.

## Discussion

### Principal findings

Comprehensive analysis of IAIHAP from 1990 to 2021, spanning 204 countries and territories, highlights six principal findings: (1) The global ASIR declined from 186.44 to 135.40 per 100,000 (AAPC: −1.74), yet IAIHAP remained a significant health challenge in 2021, with 10.5 million cases and 0.69 million YLDs. (2) Males demonstrated higher burden and more pronounced decline (AAPC: −2.58 vs −0.92 for females), particularly among those aged 15–29 years with a male-to-female ratio of 3:1. (3) High-middle SDI regions showed the largest decline (AAPC: −2.35), while low SDI regions experienced modest decreases (AAPC: −0.72) with greater fluctuation. Six of the top 10 countries with increasing burdens were in the low SDI quintile, led by Afghanistan (AAPC: 8.31) and Yemen (AAPC: 6.60), and all the top ten countries experienced wars or conflicts between 1990 and 2021. (4) Unintentional injuries dominated globally, with self-harm and interpersonal violence prevailing in low SDI regions, while higher SDI levels had more transport-related injuries and less violence. (5) Analysis based on the modeled estimates suggested that the global ASIR decline slowed during the COVID-19 pandemic compared to pre-pandemic levels, with only high SDI regions maintaining significant decreases. Of note, this finding is tentative due to data constraints during the pandemic years.

### Global burden trends and sex disparities

Between 1990 and 2021, IAIHAP demonstrated a consistent global decline, with ASIR decreasing from 186.44 to 135.40 per 100,000 population (AAPC: −1.74). Despite this overall reduction, IAIHAP remained a substantial health challenge in 2021, with over 10.5 million new cases and nearly 0.7 million YLDs globally. The decline in ASIR was accompanied by a more modest reduction in YLD rates (AAPC: −0.12), suggesting that while prevention efforts have reduced incidence, the long-term disability burden remains significant.

Sex disparities in IAIHAP burden were striking and persistent. Males showed both higher burden and more pronounced decline compared to females, with particularly wide disparities among those aged 15–29 years where the male-to-female ratio reached 3:1. This sex disparity likely reflects differences in occupational exposures, risk-taking behaviors, and participation in conflict. Notably, while acute injury rates showed marked sex differences, YLD burden demonstrated smaller sex disparities and peaked in middle adulthood for both sexes, suggesting that long-term disability impacts persist regardless of sex.

The global trends showed important variations by development level. High-middle SDI regions, despite having the highest initial burden in 1990, demonstrated the most substantial reductions. This likely benefits from better infrastructure and comprehensive safety measures, including stricter workplace regulations and traffic safety standards.^39–41^ The smoother decline curves in these regions also reflect more stable healthcare systems and societies.^42^ While higher SDI regions showed lower acute injury rates compared to lower SDI regions, they demonstrated higher ASYR, indicating longer survival with disability. This may be attributed to better medical interventions and healthcare services in these regions, which increase survival with disability, thereby contributing to the YLDs estimates.^43^

In contrast, low SDI regions experienced more modest decreases with greater fluctuation, particularly during periods of conflict or political instability. Notably, six (Afghanistan, Yemen, Rwanda, Burundi, South Sudan, and Mali) of the top ten countries with the highest ASIR-AAPC belonged to the low SDI quintile. This concentration of increasing burden in low SDI countries suggests a potential overlap of conflict and socioeconomic vulnerability. Each of these countries experienced significant periods of conflict or civil unrest during the study period. For instance, Afghanistan showed two distinct periods of increase (1990–1998 and 2003–2019) that were temporally associated with the Afghanistan Civil War and the Afghanistan War.^44,45^ Yemen’s sharp rise, particularly between 2014 and 2018, occurred concurrently with its ongoing civil war, which severely damaged healthcare infrastructure and led to widespread humanitarian crisis.^46^ Rwanda and Burundi’s high AAPC followed periods of regional conflicts and civil unrest,^47–49^ with these countries showing particularly high proportions of violence-related injuries during conflict periods (87.2% and 86.2% respectively). The remaining countries in the top ten (Libya, Congo, Armenia, and Lesotho), though not all classified as low SDI, also experienced significant political instability or conflict during the study period.^50–53^ This pattern suggests that conflict is a significant contextual factor associated with a higher injury burden regardless of baseline development level, though the impact appears most severe in settings with limited healthcare infrastructure and resources.

### Cause composition and risk distribution

The composition of IAIHAP causes showed distinct patterns across development levels. Globally, unintentional injuries dominated the IAIHAP burden in 2021, accounting for 64.6% of cases, with falls, exposure to mechanical forces, road injuries, and interpersonal violence as the leading level-three causes. These high-energy injury mechanisms are particularly significant as they frequently result in severe abdominal and pelvic trauma,^54^ often requiring extensive blood transfusion and complex medical interventions.^55^

However, the distribution of injury causes showed marked variation by socioeconomic development. A clear SDI gradient emerged in the proportion of self-harm and interpersonal violence, which was inversely correlated with development level. In low SDI regions, self-harm and interpersonal violence became predominant causes, with interpersonal violence and conflict/terrorism accounting for similar proportions (22.61% versus 22.80%). This pattern likely reflects multiple socioeconomic factors, including traditional values, lower educational attainment, and sex-related social norms characteristic of less developed regions.^26,56,57^ Moreover, economic inequality and inadequate social support systems in these regions often contribute to social instability, fostering conditions conducive to conflict and violence.^58,59^

Shifts in injury patterns were observed in several conflict-affected settings. During periods of conflict, the proportion of self-harm and interpersonal violence rose dramatically, reaching 86.0% in Syria during its civil war.^60^ This pattern was consistent across conflict-affected regions, with all ten countries showing the highest ASIR increases being significantly affected by wars between 1990 and 2021. Healthcare infrastructure is often severely compromised during conflicts; for instance, Yemen reported 120 attacks on health facilities between 2015 and 2018 during its civil war.^46^ This degradation of the healthcare system occurred alongside sharp increases in IAIHAP burden. The clinical importance of hemorrhage control in trauma care was underscored by battlefield data (2001–2011) showing that 90% of preventable combat deaths involve hemorrhage, with abdominal and pelvic regions being primary sites.^4^

In contrast, the cause patterns in higher SDI regions were dominated by transport-related injuries, reflecting their different risk profiles and more developed infrastructure. While middle SDI regions like Tonga demonstrated that low injury burden is achievable (lowest ASIR: 63.55 per 100,000) through community-based health promotion and social support systems^61,62^, the cause composition varied by development level. Higher SDI regions showed increasing proportions of transport injuries and decreasing violence-related injuries, suggesting that development level significantly influences not only the magnitude but also the nature of IAIHAP burden.

### Potential COVID-19 impacts

Analysis based on GBD model estimates suggests that the COVID-19 pandemic influenced IAIHAP burden patterns, with impacts varying by development level. The global declining trend in ASIR slowed during the pandemic (AAPC: −0.57, 95% CI: −3.31 to 2.24, 2019–2021) compared to pre-pandemic levels (AAPC: −1.56, −5.79 to 2.87, 2017–2019). Only high SDI regions maintained a significant decrease (AAPC: −0.32, *p* = 0.02), while other SDI quintiles showed non-significant changes. This pattern suggests that more developed healthcare systems were better able to maintain injury prevention and care despite pandemic disruptions.^63^ The model-based analysis also projected significant ASIR increases in specific countries, such as Guyana (AAPC: 0.63, *p* = 0.047), Lithuania (AAPC: 0.59, *p* = 0.036), and the Maldives (AAPC: 0.32, *p* = 0.018), further highlighting how pandemic impacts varied by local context and healthcare system capacity.

Analysis of the COVID-19 period was performed to understand how profound healthcare disruptions impacted non-COVID outcomes like IAIHAP. The GBD 2021 study incorporated all available data up to 2021 into its models. However, it is crucial to acknowledge that the reliability of estimates for these specific years is subject to greater uncertainty. Data completeness and timeliness were likely adversely affected in many locations due to healthcare system strain and reporting delays. Consequently, while the observed patterns, such as the slowed global decline and the resilience of high-SDI systems, are informative, they should be interpreted as the best available modeled projections based on potentially constrained data. These findings highlight the critical need for robust health information systems that can maintain surveillance during crises.

In this context, the findings reveal a paradoxical impact of the pandemic on injury patterns. While overall injury incidence decreased due to lockdown-related reductions in outdoor activities,^64^ the proportion of severe cases, including unintentional and transport injuries, increased significantly.^65,66^ This pattern likely reflects multiple factors: delayed healthcare seeking during lockdowns,^64^ changes in injury mechanisms, and potentially more severe outcomes when injuries occurred. Additionally, the biological effects of COVID-19, particularly its association with coagulation disorders,^67–69^ may have complicated injury management and increased hemorrhage risk, especially in resource-limited settings.

### Limitations and strengths

This study has several limitations. First, while the GBD study constructs cause-nature matrices from dual-coded clinical datasets to estimate specific injury outcomes, the underlying ICD coding system in source data was designed to identify the cause of injury, which can lead to incomplete characterization of the nature of injury (Supplementary Data 1). This inherent data constraint, coupled with the geographically heterogeneous availability of high-quality dual-coded data, means that estimates for regions with sparse data rely on modeling from data-rich areas. Second, the correction for individuals who sustain an injury but lack access to formal healthcare, based on the HAQ Index, is a necessary but modeled adjustment. This introduces uncertainty, as it assumes that the severity and cause-nature distribution of untreated injuries mirror those in settings with full healthcare access. Thirdly, in cases of multiple injuries, the analysis adheres to the GBD’s nature-of-injury hierarchy, which assigns the single most severe injury to each case. Consequently, the burden of less severe but concurrent injuries (like IAIHAP) may be overlooked, leading to an underestimation of their true burden. Fourthly, estimates for the pandemic period (2019–2021) are constrained by likely disruptions in healthcare utilization and reporting, resulting in greater reliance on model extrapolation and increased uncertainty. Finally, a data inconsistency in the GBD 2021 dataset is observed, where the sum of the ASIR of level 3, causes under “unintentional injuries”, does not equal the total ASIR for “unintentional injuries” overall, despite being expected to match.

Despite these challenges, this study provides a comprehensive analysis of the global burden of IAIHAP, with coverage of 204 countries and territories, assessment of trends over three decades, and detailed evaluation of socioeconomic and geographic patterns, thereby providing crucial insights into this previously underexplored aspect of trauma care. By analyzing IAIHAP patterns across diverse conflict settings, this study characterized the injury burden within these vulnerable contexts. This study allows for robust trend identification and highlights key disparities by development level, age, and sex. Integrating timelines of instability with injury estimates offers descriptive insights into the co-occurrence of political crises and population-level injury patterns. The inclusion of COVID-19 impact analysis further enhances the understanding of how global health crises shape injury patterns across different development levels. In light of these findings, several policy considerations should be considered. In conflict-affected and other fragile settings, priorities include investing in resilient health systems, protecting healthcare infrastructure from attacks, and strengthening pre-hospital and emergency medical services to maintain trauma care capacity during crises. Developing trauma care pathways that remain functional during public health emergencies is also essential to ensure continuity of care.

Future research should prioritize cross-country comparisons of trauma care systems to identify policy and infrastructure factors contributing to burden variation. Mechanistic studies on how systemic inflammation worsens hemorrhage risk may also inform targeted interventions. Additionally, evaluating emerging strategies, such as improved hemorrhage control methods or decision-support tools, could help enhance trauma care, particularly in settings affected by conflict. Although these areas extend beyond the scope of the current study, the identification of scalable and context-appropriate solutions remains critical to reducing the global burden of IAIHAP.

## Conclusion

This study demonstrates that while global IAIHAP burden has declined over the past three decades, significant disparities persist across regions, development levels, and demographic groups. Despite the overall global decline in incidence and disability rates, IAIHAP remains a critical public health challenge, especially in low-SDI regions where conflict and political instability exacerbate its impact. The findings emphasize the need for tailored interventions that address both the direct medical challenges and the socio-political factors driving disparities.

## Supplementary information

Transparent Peer Review file

Supplementary information

Description of Additional Supplementary files

Supplementary Data 1

Supplementary Data 2

Supplementary Data 3

Supplementary Data 4

Supplementary Data 5

Supplementary Data 6

Supplementary Data 7

Supplementary Data 8

Supplementary Data 9

Supplementary Data 10

## Data Availability

The datasets analyzed during the current study are publicly available and were obtained from the Global Burden of Diseases, Injuries, and Risk Factors Study (GBD) database. These datasets can be accessed through the following link: (https://vizhub.healthdata.org/gbd-results/). Free user registration may be required to access certain datasets. The numerical data underlying the figures are provided in the Supplementary Data files: The source data for Fig. 1 are in Supplementary Data 2 and 8; The source data for Fig. 2 are in Supplementary Data 9. The source data for Fig. 3 are in Supplementary Data 10. The source data for Fig. 4 are in Supplementary Data 2. The map images in article were created using the rnatural earth R package, which provides access to Natural Earth map data. This data is open-source and available at http://github.com/ropensci/rnaturalearth.
