# Supplementary material for: Global, regional, and national burden of injury associated internal hemorrhage in the abdomen and pelvis from 1990-2021
Source: Commun Med (Lond). 2026 Apr 13;6:431. doi: 10.1038/s43856-026-01560-3 (PMC13444077; doi:10.1038/s43856-026-01560-3)
Supplement: Supplementary file 1 [file 43856_2026_1560_MOESM1_ESM.pdf]

nature portfolio

Peer Review File

Global, regional, and national burden of injury associated internal hemorrhage in the abdomen and pelvis from 1990-2021

Corresponding Author: Professor Ting Luo

This file contains all reviewer reports in order by version, followed by all author rebuttals in order by version.

Version 0:

Reviewer comments:

Reviewer #1

(Remarks to the Author)
The authors investigate injury-associated internal hemorrhage in the abdomen and pelvis (IAIHAP) using data from the Global Burden of Disease (GBD) Study 2021. Their analysis spans three decades (1990–2021), providing detailed incidence rates, YLDs, and trends stratified by age, sex, region, and socio-demographic index. They also employ modeling techniques to project the future global incidence of IAIHAP up to 2035. This paper holds promise for informing public health strategies relevant to trauma and emergency care worldwide, especially in conflict-affected and low-resource settings.

Comments:

Although the manuscript highlights the importance of preventing avoidable deaths from IAIHAP, the rationale would benefit from the inclusion of more recent and comprehensive literature. Emphasizing recent research on improvements in trauma management, the global burden of conflict-related injuries, and related public health policies will strengthen the motivations for conducting this study.

Please provide additional details on the methodology used within the GBD framework. Discuss the data sources, model assumptions, and analytical tools integral to GBD estimates. This will help readers fully comprehend the robustness and complexity associated with GBD data analysis.

While the manuscript presents numerous statistics, it would be beneficial to include a clear explanation of how CIs were calculated and how they should be interpreted in the context of burden estimates. This will reinforce the credibility of the statistical methods and outcomes.

In addition to confidence intervals, the authors refer to uncertainty intervals in the projections. Please clarify how these UIs were calculated, their rationale, and differences (if any) from the CIs. This will help the readership interpret the range of potential future burdens correctly.

It would be helpful to explicitly discuss the implications of this study’s findings for public health strategies. Identify any actionable recommendations—such as policy changes, targeted interventions, or resource allocations—that could meaningfully reduce the burden of IAIHAP.

The manuscript should maintain a consistent format for decimal representation throughout. Decide on either one decimal place (e.g., 0.0) or two decimal places (e.g., 0.00) and apply it uniformly to improve the clarity and professionalism of the presentation.

In some sections, non-significant findings are presented without thorough justification or discussion. Consider removing these findings or clarifying their relevance to the study’s key messages if they add context (e.g., potential trends or directions for further research).

The manuscript would benefit from a dedicated section highlighting future research opportunities. For instance, you may

suggest the exploration of novel interventions, cross-country comparative studies of trauma systems, or refined modeling techniques that might better capture regional variations and conflict-related factors.

Reviewer #2

(Remarks to the Author)
This reviewer's most serious objections is the forecasting or trajectories component. While, for example, forecasting cancer morbidity and mortality makes sense because cancer is caused by long term stable trends: aging of populations, environmental, stable life style habits and the like. Also for Type 2 diabetes prevalence, forecasting or trajectories can be useful, since obesity and nutrition develop decades before symptoms. Injuries, in contrast, are unpredictable. Who could have forecasted that 2% of all Russian males 18-50 years of age died or were seriously wounded in the war against the Ukraine?2022-2024?
Thus, drop all according sections, including the high-level equations. This reviewer has a university level training in mathematics, so it is not any prejudice.

Otherwise, a useful informative report, very well worth to be published.

Details
• P 03: "Notably, injury-associated hemorrhage is a major contributor to "preventable deaths" (potentially survivable patients who ultimately died), with over two-thirds of lethal bleeding occurring in the truck,3 and 64% of such truncal injuries specifically located in the abdomen and pelvis." Do you mean trunk?
• P 03 "While blunt trauma can directly dagame pelvic organs or soft tissues (including bladder, uterus, gonads), most cases of injury-associated internal pelvis hemorrhage stem from pelvic fractures" read "damage"
• P 03 "The relationship between pelvic and abdominal injuries is notably interconnected" This is a unnecessary hendiadyoin
• P 04 "This study utilizes a retrospective cohort design to analyze data derived from the Global Burden of Disease, Injuries, and Risk Factors Study (GBD) 2021, which provides comprehensive estimates of disease burden across 371 diseases and injuries, including 40 injury categories" This is not a retrospective cohort designs, it is a retrospective trend design. GBD uses aggregate data.
• P06 the equation overall trend: AAPC = (exp($\Sigma wibi \Sigma wi$) - 1) x 100 – especially the term in brackets – Is insufficiently explained.
• P06-p07: Forecasting –main objections: forecasting cancer morbidity and mortality, for example, makes sense because cancer is caused by long term stable trends: aging of populations, environmental, stable life style habits and the like. Injuries are unpredictable, instead. Who could have forecasted that 2% of all Russian males 18-50 years of age died or were seriously wounded in the war against the Ukraine?2022-2024
• P13-p14 "Principal findings" Relevant and superbly written!
• P16-p17 "COVID-19 Impact and Future Trajectories" COVID19 very important, Trajectories pure speculations!

Version 1:

Reviewer comments:

Reviewer #1

(Remarks to the Author)
This manuscript presents a detailed epidemiological analysis of the burden of IAIHAP from 1990 to 2021 by leveraging the comprehensive data from the Global Burden of Disease (GBD) Study 2021. The authors provide robust estimations of incidence, prevalence, and years lived with disability (YLDs) across 204 countries and territories. The analysis further stratifies findings by sex, age, region, and socio-demographic index (SDI), with temporal trends evaluated via join-point regression methods. The study offers valuable insight into the shifting global patterns of IAIHAP burden, highlighting important regional disparities, particularly with respect to conflict-affected and low-resource settings, underscoring the need for tailored public health interventions.

I find this manuscript suitable for publication in the journal

Reviewer #3

(Remarks to the Author)
Thanks to the authors for writing on this topic and for affording me the opportunity to review. The manuscript highlights oft under-utilized estimates from GBD and identifies important trends and limitations of the current estimates. Additionally the authors have responded well to the initial round of reviewer feedback. However, crucial areas for improvement remain in terms of accuracy and clarity around GBD data sources and methodology. The comments I have provided seek to offer guidance in this regard.
1. Please review relevant papers and resources describing GBD methods and data sources and update the methods section accordingly. Certain points are not accurate and/or not detailed enough for the reader to understand the processes in generating these estimates. Specific examples are highlighted below.
a. Please describe how the nature of injury estimates are generated. The modeling tool described, DisMod-MR, is only

utilized to estimate causes of injury. The manuscript currently does not touch on the data sources or methods used to further estimate specific natures of injury, such as internal hemorrhage. Lines 129-136 are insufficient in this regard. A specific publication that may assist: James SL, Castle CD, Dingels ZV, et al. Estimating global injuries morbidity and mortality: methods and data used in the Global Burden of Disease 2017 study. Injury Prevention 2020;26:i125-i153.
b. Lines 87-88: Where did the number 4,722 hospital discharge records across 49 countries come from? Is this meant to describe GBD in general, or specifically internal hemorrhage? As written, it is not correct that clinical data was available for 49 countries for this specific topic in GBD analysis. Please investigate and clarify.
c. Lines 91-92: Please confirm whether these claims databases were actively used to inform estimates of internal hemorrhage. If not, consider removing this detail for clarity.
d. Are there other types of data (outside of administrative and clinical data, and the conflict databases)? Please clarify and provide examples.
e. The databases providing information related to conflict (e.g., Uppsala) are primarily used to estimate mortality. Please justify or clarify the purpose of including this list of sources in the manuscript, when its focus is on non-fatal injury outcomes.
f. Please revise the section on limitations of GBD after updating the methods, as described above. It is not correct that GBD only uses ICD codes to identify cause of injury (line 419), and it is also incorrect that GBD lacks cause-nature pair data entirely (line 424). There are other key limitations of data for IAIHAP in GBD analyses (and methodological challenges) that are not mentioned here.
2. Please justify the use of methods to separately calculate AAPCs when GBD already produces estimates of percent change and provides them via the GBD Results Tool and GBD Compare.
3. When reporting GBD results, please always include the 95% uncertainty interval (UI) and label it as such. This was not consistently done in the Results section.
4. Please provide a detailed justification for analyzing trends during the COVID-19 pandemic. Is there data available to support the estimates and conclusions drawn in the text (i.e., was there input data to GBD modeling processes during these years)? Discuss the reliability and limitations of findings for the pandemic years specifically.
5. Please clarify where the percentage estimates in the Results section titled “The cause proportion of IAIHAP” came from – are these GBD estimates, or were they calculated by the authors? If GBD estimates, please provide the 95% UIs accompanying. If calculated by the authors, please clarify.
6. Please justify your focus on countries experiencing conflict. Are observed trends supported by underlying data or primarily driven by modeling assumptions? Avoid statements implying causality between conflict and IAIHAP burden, as this is not within the scope of GBD. The manuscript often reads as though the estimates presented are primarily driven by conflict-related injuries, which is misleading.
7. The introduction places significant emphasis on pelvic fractures, yet the manuscript focuses on IAIHAP exclusively. GBD makes estimates of pelvic fractures separately from IAIHAP. Please revise the introduction for consistency with the main analysis. If pelvic fractures are not analyzed, avoid highlighting them in the introduction.
8. Please avoid using “our” when referring to GBD (e.g., lines 422 and 427) unless you contributed to GBD estimates as GBD collaborators. This distinction ensures clarity about your role versus those of the GBD researchers and those who are part of the GBD Collaborator Network.

Version 2:

Reviewer comments:

Reviewer #3

(Remarks to the Author)
Thank you to the authors for their thoughtful revisions and for addressing many of the previous comments. The manuscript is much improved, particularly with respect to the clarity and detail provided in the methods section.

However, one item remains insufficiently addressed: the section discussing trends during the COVID-19 pandemic. Please clarify the amount of data available for the years 2019–2021 that informed the modeled estimates. My concern is that there may be very limited (or no) input data for these years, which would mean that conclusions are primarily model-driven. To strengthen this section, the authors could either (a) provide specific information on the number and type of data sources available for this three-year period, and strongly emphasize that these results should be interpreted with caution, or (b) consider removing commentary on country-specific trends during the pandemic.

Version 3:

Reviewer comments:

Reviewer #3

(Remarks to the Author)
Thanks to the authors for their consideration and incorporation of the feedback I have shared. The revisions are important and have addressed my outstanding concerns.

Open Access This Peer Review File is licensed under a Creative Commons Attribution 4.0 International License, which permits use, sharing, adaptation, distribution and reproduction in any medium or format, as long as you give appropriate credit to the original author(s) and the source, provide a link to the Creative Commons license, and indicate if changes were made.
In cases where reviewers are anonymous, credit should be given to 'Anonymous Referee' and the source.
The images or other third party material in this Peer Review File are included in the article's Creative Commons license, unless indicated otherwise in a credit line to the material. If material is not included in the article's Creative Commons license and your intended use is not permitted by statutory regulation or exceeds the permitted use, you will need to obtain permission directly from the copyright holder.
To view a copy of this license, visit https://creativecommons.org/licenses/by/4.0/

ID: COMMSMED-25-0505-T
Title: Global, regional, and national burden of injury associated internal hemorrhage in abdomen and pelvis from 1990 to 2021: findings from the global burden of disease study 2021

Reviewer #1 (Remarks to the Author):

The authors investigate injury-associated internal hemorrhage in the abdomen and pelvis (IAIHAP) using data from the Global Burden of Disease (GBD) Study 2021. Their analysis spans three decades (1990–2021), providing detailed incidence rates, YLDs, and trends stratified by age, sex, region, and socio-demographic index. They also employ modeling techniques to project the future global incidence of IAIHAP up to 2035. This paper holds promise for informing public health strategies relevant to trauma and emergency care worldwide, especially in conflict-affected and low-resource settings.

Comments:

1. Although the manuscript highlights the importance of preventing avoidable deaths from IAIHAP, the rationale would benefit from the inclusion of more recent and comprehensive literature. Emphasizing recent research on improvements in trauma management, the global burden of conflict-related injuries, and related public health policies will strengthen the motivations for conducting this study.

Response: We thank the reviewer for this insightful and constructive comment. We have updated the Introduction section to incorporate recent conflicts, highlighting their impact on healthcare systems to enhance the study's relevance and urgency. We also added recent literature on trauma management, emphasizing the importance of controlling abdominopelvic hemorrhage to reduce mortality risk (page 4, lines 59-68).

2. Please provide additional details on the methodology used within the GBD framework. Discuss the data sources, model assumptions, and analytical tools integral to GBD estimates. This will help readers fully comprehend the robustness and complexity associated with GBD data analysis.

Response: Thank you for the useful suggestion. We have updated the relevant content to provide a more detailed introduction to data sources, model assumptions, and analytical tools (page 5, lines 85-102; pages 6, lines 129-136)

3. While the manuscript presents numerous statistics, it would be beneficial to include a clear explanation of how CIs were calculated and how they should be interpreted in the context of burden estimates. This will reinforce the credibility of the statistical methods and outcomes.

Response: We thank the reviewer for this insightful comment. In response, we have provided a clear explanation of how CIs were calculated and elucidated their role in

predicting disease trends (page 7-8, lines 160-168).

4. In addition to confidence intervals, the authors refer to uncertainty intervals in the projections. Please clarify how these UIs were calculated, their rationale, and differences (if any) from the CIs. This will help the readership interpret the range of potential future burdens correctly.

Response: Thank you for your valuable suggestion. As you point out, describing the methods for calculating UIs could indeed help the readership understand the disease burden estimates. However, given the complexity and multifaceted nature of how the GBD study calculates UIs, we prefer not to provide a detailed description within the main text. Instead, we will strategically include key references at pertinent points in the manuscript, which will allow interested readers to explore the comprehensive methodological details (page 4, lines 81-83). For context, the following provides a concise overview of the UI calculation process used: Uncertainty in claims estimates was calculated using Wilson's approximation, utilizing sample size derived from enrollment data (i.e. Marketscan) or GBD population estimates (i.e. Poland), depending on the source. Uncertainty in outpatient estimates was also calculated using Wilson's approximation and GBD population. Uncertainty for inpatient sources that are not complete for the population and use the inpatient utilization envelope came from the upper and lower uncertainty intervals of 1000 bootstrapped samples of the envelope and correction factor models. Inpatient sources that are complete for the population derived uncertainty from Wilson's approximation and GBD population. Wilson's approximation:

$$\sigma^2 = \frac{\frac{cf(1-cf)}{n} + \frac{1.96^2}{4n^2}}{\left(1 + \frac{1.96^2}{n}\right)^2}$$

Tips： *cf* is the cause fraction and *n* is the sample size

The Lower 95% UI is the 2.5th percentile of estimates. The Upper 95% UI is the 97.5th percentile of estimates.

5. It would be helpful to explicitly discuss the implications of this study's findings for public health strategies. Identify any actionable recommendations—such as policy changes, targeted interventions, or resource allocations—that could meaningfully reduce the burden of IAIHAP.

Response: We agree with this valuable perspective. As highlighted, quantifying the burden is only the first step, translating these findings into actionable strategies would improve public health outcomes. We have added a dedicated paragraph in the Discussion section outlining several public health considerations informed by our findings (page 18, lines 441-448;). These include strategies such as integrating basic emergency medical training into community rebuilding efforts, promoting resilient trauma care systems, and enhancing awareness of inflammation-related risks in trauma outcomes. However, given the inherent limitations of our retrospective ecological study design, we have been careful not to overextend the implications beyond the scope of our data. Instead, we have framed these points as preliminary considerations that may inform future research and policy discussions, rather than definitive recommendations.

6. The manuscript should maintain a consistent format for decimal representation throughout. Decide on either one decimal place (e.g., 0.0) or two decimal places (e.g., 0.00) and apply it uniformly to improve the clarity and professionalism of the presentation.

Response: Thank you for this insightful suggestion. We have addressed this issue by standardizing decimal presentation, increasing precision to two decimal places throughout the manuscript.

7. In some sections, non-significant findings are presented without thorough justification or discussion. Consider removing these findings or clarifying their relevance to the study's key messages if they add context (e.g., potential trends or directions for further research).

Response: Thank you for this valuable feedback. We have revised the manuscript by removing certain findings as suggested and expanding the discussion with targeted analysis.

8. The manuscript would benefit from a dedicated section highlighting future research opportunities. For instance, you may suggest the exploration of novel interventions, cross-country comparative studies of trauma systems, or refined modeling techniques that might better capture regional variations and conflict-related factors.

Response: We thank the reviewer for this constructive suggestion. We have added a paragraph in the Discussion section outlining future research opportunities, including cross-country comparisons of trauma systems, exploration of novel intervention strategies, and refined modeling approaches to better capture regional disparities and conflict-related influences (page 18, lines 449-455).

Reviewer #2 (Remarks to the Author):

This reviewer's most serious objections is the forecasting or trajectories component. While, for example, forecasting cancer morbidity and mortality makes sense because cancer is caused by long term stable trends: aging of populations, environmental, stable life style habits and the like. Also for Type 2 diabetes prevalence, forecasting or trajectories can be useful, since obesity and nutrition develop decades before symptoms. Injuries, in contrast, are unpredictable. Who could have forecasted that 2% of all Russian males 18-50 years of age died or were seriously wounded in the war against the Ukraine?2022-2024?
Thus, drop all according sections, including the high-level equations. This reviewer has a university level training in mathematics, so it is not any prejudice.
Otherwise, a useful informative report, very well worth to be published.
Response: Thank you for this insightful suggestion. As recommended, we have removed all forecasting related content. This adjustment better conforms to the epidemiological characteristics of injuries and enhances the rigor of this article.

Details

1. P 03: "Notably, injury-associated hemorrhage is a major contributor to "preventable deaths" (potentially survivable patients who ultimately died), with over two-thirds of lethal bleeding occurring in the truck,3 and 64% of such truncal injuries specifically located in the abdomen and pelvis." Do you mean trunk? P 03 "While blunt trauma can directly dagame pelvic organs or soft tissues (including bladder, uterus, gonads), most cases of injury-associated internal pelvis hemorrhage stem from pelvic fractures" read "damage"

Response: We thank the reviewer for pointing out these typographical errors. We have revised the manuscript accordingly (page 3, line 37, 43). We have also conducted a thorough proofreading of the manuscript to ensure terminological accuracy and improve overall clarity and consistency.

2. P03 "The relationship between pelvic and abdominal injuries is notably interconnected" This is a unnecessary hendiadyoin

Response: Thank you for this insightful suggestion. We have revised the sentence to eliminate the redundant phrasing, thereby improving the conciseness and clarity of the manuscript (page , line 53).

3. P 04 "This study utilizes a retrospective cohort design to analyze data derived from the Global Burden of Disease, Injuries, and Risk Factors Study (GBD) 2021, which provides comprehensive estimates of disease burden across 371 diseases and injuries, including 40 injury categories" This is not a retrospective cohort designs, it is a retrospective trend design. GBD uses aggregate data.

Response: We thank the reviewer for identifying this important clarification and have revised text accordingly (page 4, line 80).

4. P06 the equation overall trend: AAPC = (exp($\Sigma wibi\Sigma wi$) - 1) x 100 – especially the term in brackets – Is insufficiently explained.

Response: We thank the reviewer for this constructive suggestion. We have added detailed explanations to the text in brackets (page 7, lines 153-155).

5. P06-p07: Forecasting －main objections: forecasting cancer morbidity and mortality, for example, makes sense because cancer is caused by long term stable trends: aging of populations, environmental, stable life style habits and the like. Injuries are unpredictable, instead. Who could have forecasted that 2% of all Russian males 18-50 years of age died or were seriously wounded in the war against the Ukraine?2022-2024

Response: Thank you for this insightful suggestion. We have removed all forecasting components from the manuscript. This revision ensures the study remains aligned with the inherently unpredictable nature of injury-related events and strengthens the overall methodological appropriateness and rigor of the analysis.

6. P13-p14 “Principal findings” Relevant and superbly written!

Response: We sincerely appreciate the editor’s recognition of the principal findings section.

7. P16-p17 “COVID-19 Impact and Future Trajectories” COVID19 very important, Trajectories pure speculations!

Response: Thank you for this insightful suggestion. We have removed the “Future Trajectories” subsection to maintain focus on evidence-based findings and avoid speculative content.

ID: COMMSMED-25-0505A
Title: Global, regional, and national burden of injury associated internal hemorrhage in abdomen and pelvis from 1990 to 2021: findings from the global burden of disease study 2021

Reviewer #1 (Remarks to the Author):

Comment: This manuscript presents a detailed epidemiological analysis of the burden of IAIHAP from 1990 to 2021 by leveraging the comprehensive data from the Global Burden of Disease (GBD) Study 2021. The authors provide robust estimations of incidence, prevalence, and years lived with disability (YLDs) across 204 countries and territories. The analysis further stratifies findings by sex, age, region, and socio-demographic index (SDI), with temporal trends evaluated via join-point regression methods. The study offers valuable insight into the shifting global patterns of IAIHAP burden, highlighting important regional disparities, particularly with respect to conflict-affected and low-resource settings, underscoring the need for tailored public health interventions.
I find this manuscript suitable for publication in the journal.

Response: Thank you for your positive and encouraging feedback on our manuscript. We are delighted to hear that the reviewer finds our work suitable for publication.

Reviewer #3 (Remarks to the Author):

Comment 1: Thanks to the authors for writing on this topic and for affording me the opportunity to review. The manuscript highlights the under-utilized estimates from GBD and identifies important trends and limitations of the current estimates. Additionally, the authors have responded well to the initial round of reviewer feedback. However, crucial areas for improvement remain in terms of accuracy and clarity around GBD data sources and methodology. The comments I have provided seek to offer guidance in this regard.

Please review relevant papers and resources describing GBD methods and data sources and update the methods section accordingly. Certain points are not accurate and/or not detailed enough for the reader to understand the processes in generating these estimates. Specific examples are highlighted below.
a. Please describe how the nature of injury estimates are generated. The modeling tool described, DisMod-MR, is only utilized to estimate causes of injury. The manuscript currently does not touch on the data sources or methods used to further estimate specific natures of injury, such as internal hemorrhage. Lines 129-136 are insufficient in this regard. A specific publication that may assist: James SL, Castle CD, Dingels ZV, et al. Estimating global injuries morbidity and mortality: methods and data used in the Global Burden of Disease 2017 study. Injury Prevention 2020;26:i125-i153.

Response 1a: Thank you for this insightful comment and for pointing out the lack of detail in method section. In response, we have revised the Methods section (line 78-100) to provide a detailed account for estimating the nature of injury process. Meanwhile, relevant references have been added as suggested (citation 27-28).

Comment 1b: Lines 87-88: Where did the number 4,722 hospital discharge records across 49 countries come from? Is this meant to describe GBD in general, or specifically internal hemorrhage? As written, it is not correct that clinical data was available for 49 countries for this specific topic in GBD analysis. Please investigate and clarify.

Response 1b: We thank the reviewer for pointing out this inaccuracy. Upon thorough investigation, we confirmed this context did not accurately represent the data sources for this specific analysis. To avoid potential misunderstanding and ensure methodological accuracy, we have removed this sentence from the text. We believe this correction improves the precision and clarity of our methods description.

Comment 1c: Lines 91-92: Please confirm whether these claims databases were actively used to inform estimates of internal hemorrhage. If not, consider removing this detail for clarity.

Response 1c: We thank the reviewer for the suggestion. The mentioned text has been removed to improve clarity.

Comment 1d: Are there other types of data (outside of administrative and clinical data, and the conflict databases)? Please clarify and provide examples.

Response 1d: We thank the reviewer for raising this point. We have now clarified the inclusion of additional data types in the revised manuscript (line96-100). Specifically, we have added that population-representative surveys (such as the WHS and MEPS) and supplementary sources (including collaborator accounts and news reports) to ensure comprehensive coverage.

Comment 1e: The databases providing information related to conflict (e.g., Uppsala) are primarily used to estimate mortality. Please justify or clarify the purpose of including this list of sources in the manuscript, when its focus is on non-fatal injury outcomes.

Response 1e: We thank the reviewer for raising this important point. The inclusion of these conflict and disaster databases is indeed critical for estimating non-fatal outcomes in the context of fatal discontinuities. In GBD 2021, direct incidence data for these events are largely unavailable, therefore, an incidence-to-mortality ratio approach is applied to derive non-fatal injury incidence from mortality estimates. We have revised the text accordingly (line 101-108).

Comment 1f: Please revise the section on limitations of GBD after updating the methods, as described above. It is not correct that GBD only uses ICD codes to identify cause of injury (line 419), and it is also incorrect that GBD lacks cause-nature pair data entirely (line 424). There are other key limitations of data for IAIHAP in GBD analyses (and methodological challenges) that are not mentioned here.

Response 1f: We thank the reviewer for pointing out the inaccuracies in limitations section. We have revised this section. The incorrect statements regarding the exclusive use of ICD

codes for cause of injury and the complete lack of cause-nature pair data have been removed. In addition, we have also expanded the limitations provide a more balanced reflection of methodological challenges.

Specifically, we now emphasize that, first, uneven data quality and completeness across countries may lead to regional estimates that do not fully capture local injury patterns. Second, the adjustment using the Healthcare Access and Quality (HAQ) Index to model untreated injuries introduces uncertainty, since it assumes that the severity and cause–nature profile of untreated cases resemble those receiving care. Third, the GBD nature-of-injury hierarchy, which assigns only the most severe injury per case, may underestimate the contribution of concurrent yet clinically important injuries such as IAIHAP. Finally, we note an internal data inconsistency identified within the GBD 2021 dataset, where aggregated values across sub-causes did not perfectly align with category totals. These revisions appear in the updated manuscript (line 450-462)

Comment 2: Please justify the use of methods to separately calculate AAPCs when GBD already produces estimates of percent change and provides them via the GBD Results Tool and GBD Compare.

Response 2: We thank the reviewer for this valuable question, which allowed us to clarify the rationale for employing Joinpoint regression to calculate the Average Annual Percentage Change (AAPC). Unlike the percentage change estimates provided by GBD, the AAPC offers a model-based summary measure that accounts for year-to-year fluctuations and provides confidence intervals for statistical inference .[1] This approach enables a more robust assessment of trend magnitude and direction over extended time periods and facilitates standardized comparisons of change rates across regions and causes. We have clarified this justification in the revised Methods section (lines 162–167).

Comment 3: When reporting GBD results, please always include the 95% uncertainty interval (UI) and label it as such. This was not consistently done in the Results section.

Response 3: We thank the reviewer for this comment. In line with standard GBD reporting conventions, we report 95% uncertainty intervals (UIs) at the first mention of each measure but do not repeat them throughout the text to maintain clarity and conciseness. This format follows the GBD Capstones.[2-4]

Comment 4: Please provide a detailed justification for analyzing trends during the COVID-19 pandemic. Is there data available to support the estimates and conclusions drawn in the text (i.e., was there input data to GBD modeling processes during these years)? Discuss the reliability and limitations of findings for the pandemic years specifically.

Response 4: We thank the reviewer for this important comment. We have revised the manuscript to justify the inclusion of the COVID-19 period and clarify data reliability. Specifically, we now emphasize the relevance of assessing how healthcare disruptions affected non-COVID injury outcomes and note that GBD 2021 incorporated all available data up to 2021. A dedicated discussion has been added to acknowledge the greater uncertainty of estimates during the pandemic due to potential data incompleteness and reporting delays. We

further contextualized these findings with evidence on shifts in injury patterns and possible biological mechanisms influencing outcomes. (line 432-447)

Comment 5: Please clarify where the percentage estimates in the Results section titled “The cause proportion of IAIHAP” came from – are these GBD estimates, or were they calculated by the authors? If GBD estimates, please provide the 95% UIs accompanying. If calculated by the authors, please clarify.

Response 5: We thank the reviewer for raising this question. The revised manuscript has clarified that the percentage estimates were calculated by the authors by dividing the cause-specific ASR by the overall ASR for all injuries, based on the GBD 2021 point estimates (line 156-159). As these proportions were derived directly from point estimates rather than from modeled draws, corresponding uncertainty intervals were not separately presented.

Comment 6: Please justify your focus on countries experiencing conflict. Are observed trends supported by underlying data or primarily driven by modeling assumptions? Avoid statements implying causality between conflict and IAIHAP burden, as this is not within the scope of GBD. The manuscript often reads as though the estimates presented are primarily driven by conflict-related injuries, which is misleading.

Response 6: We thank the reviewer for this valuable and constructive comment. We have carefully revised the manuscript to clarify the rationale for focusing on countries experiencing conflict. The intention of this analysis was not to infer causality but to describe observed patterns in IAIHAP burden in relation to contextual factors, including conflict exposure.

We have now explicitly stated that the observed increases in conflict-affected countries are based on modelled estimates informed by available mortality and injury data, and that such trends may also reflect limitations in data availability and modelling assumptions in these settings. To avoid any implication of causality, statements suggesting a direct effect of conflict on IAIHAP burden have been rephrased to emphasize correlation rather than causation. These clarifications are now incorporated into the Discussion section (lines 372-385).

Comment 7: The introduction places significant emphasis on pelvic fractures, yet the manuscript focuses on IAIHAP exclusively. GBD makes estimates of pelvic fractures separately from IAIHAP. Please revise the introduction for consistency with the main analysis. If pelvic fractures are not analyzed, avoid highlighting them in the introduction.

Response 7: We thank the reviewer for this comment. The introduction has been revised to align with our focus on IAIHAP by reframing the narrative away from pelvic fractures specifically. These are now presented as a representative example of severe IAIHAP rather than a central topic, ensuring consistency with the main analysis.

Comment 8: Please avoid using “our” when referring to GBD (e.g., lines 422 and 427) unless you contributed to GBD estimates as GBD collaborators. This distinction ensures clarity about your role versus those of the GBD researchers and those who are part of the GBD Collaborator Network.

Response 8: We appreciate the reviewer’s careful attention to this distinction. As members of

the GBD Collaborator Network, we have contributed input during the circulation and internal review stages. Therefore, the use of "our" in referring to GBD estimates was intended to acknowledge this collaborative involvement. Nevertheless, to ensure absolute clarity for readers, we have revised the phrasing to refer to "the GBD study" or "GBD estimates" directly where appropriate (lines 113-114, 450, 498).

Reference

1. Clegg LX, Hankey BF, Tiwari R, Feuer EJ, Edwards BK. Estimating average annual per cent change in trend analysis. *Statistics in medicine.* 2009;28(29):3670-3682.
2. Global, regional, and national burden of household air pollution, 1990-2021: a systematic analysis for the Global Burden of Disease Study 2021. *Lancet (London, England).* 2025;405(10485):1167-1181.
3. Global, regional, and national prevalence of adult overweight and obesity, 1990-2021, with forecasts to 2050: a forecasting study for the Global Burden of Disease Study 2021. *Lancet (London, England).* 2025;405(10481):813-838.
4. Global, regional, and national prevalence of child and adolescent overweight and obesity, 1990-2021, with forecasts to 2050: a forecasting study for the Global Burden of Disease Study 2021. *Lancet (London, England).* 2025;405(10481):785-812.
