# Supplementary material for: Global, regional, and national burden of injury associated internal hemorrhage in the abdomen and pelvis from 1990-2021
Source: Commun Med (Lond). 2026 Apr 13;6:431. doi: 10.1038/s43856-026-01560-3 (PMC13444077; doi:10.1038/s43856-026-01560-3)
Supplement: Supplementary file 2 [file 43856_2026_1560_MOESM2_ESM.pdf]

Supplementary figure/table legends:

Figure S1: Changes for injury associated internal hemorrhage in abdomen and pelvis (IAIHAP) burden in top ten countries with AAPC of ASIR.

(A) Afghanistan (B) Yemen (C) Rwanda (D) Libya (E) Burundi (F) South Sudan

(G) Congo (H) Armenia (I) Mali (J) Lesotho.

AAPC: average annual percent change; ASIR: age-standardized incidence rate

Figure S2: Changes for injury associated internal hemorrhage in abdomen and pelvis (IAIHAP) burden in top ten countries with AAPC of ASYR.

(A) Rwanda (B) Syria (C) Libya (D) Burundi (E) Yemen (F) Lesotho (G) South Sudan

(H) Papua New Guinea (I) Belize (J) Afghanistan (K) Botswana.

AAPC, average annual percent change; Age-standardized years lived with disability (YLDs) rate (ASYR).

Figure S3: The correlation between Injury associated internal hemorrhage in abdomen and pelvis (IAIHAP) burden and SDI value in quintiles statistics significance in 2021.

(A) Age-standardized incidence rate (ASIR) in high SDI quintile region;

(B) ASIR in low-middle SDI quintile region;

(C) Age-standardized years lived with disability (YLDs) rate (ASYR) in low-middle SDI quintile region.

ASIR: age-standardized incidence rate.

Table S1: Level two/three cause proportion of ASIR of injury-associated internal

hemorrhage in abdomen and pelvis (IAIHAP) in global and 5 SDI quintiles regions, 2021.

ASIR, age-standardized-incidence-rate; SDI, sociodemographic index.

Table S2: Level two/three cause proportion of ASYR of injury-associated internal hemorrhage in abdomen and pelvis (IAIHAP) in global and 5 SDI quintiles regions, 2021. ASYR, age-standardized-YLDs-rate; SDI, sociodemographic index; YLDs, years lived with disability.

Table S3: Cause proportion of ASIR of injury-associated internal hemorrhage in abdomen and pelvis (IAIHAP) in GBD region, 2021.

ASIR, age-standardized-incidence-rate; SDI, sociodemographic index.

Table S4: Cause proportion of ASYR of injury-associated internal hemorrhage in abdomen and pelvis (IAIHAP) in GBD region, 2021.

ASYR, age-standardized-YLDs-rate; SDI, sociodemographic index; YLDs, years lived with disability.

Table S5: Cause proportion of top 10 countries/regions with highest AAPC of ASIR, non-peaceful period.

AAPC, average annual percent change; ASIR, age-standardized-incidence-rate; SDI, sociodemographic index.

Table S6: Cause proportion of top 10 countries/regions with highest AAPC of ASIR, the whole time.

AAPC, average annual percent change; ASIR, age-standardized-incidence-rate; SDI, sociodemographic index.

Table S7: The correlation between ASIR/ASYR and SDI in global and 5 SDI quintiles regions in 2021.

ASIR, age-standardized-incidence-rate; ASYR, age-standardized-YLDs-rate; SDI, sociodemographic index; YLDs, years lived with disability. Two-sided t-tests are used for statistical test, with no adjustment for multiple comparisons. The exact P-value of the correlation is shown below:

	SDI-ASIR	SDI-ASYR
Global	2.9907E-7	3.2352E-14
High SDI	0.000008	0.219405
High-middle SDI	0.139107	0.089594
middle SDI	0.141537	0.511919
Low-middle SDI	0.002047	0.000403
Low SDI	0.117779	0.607919

Figure S1:

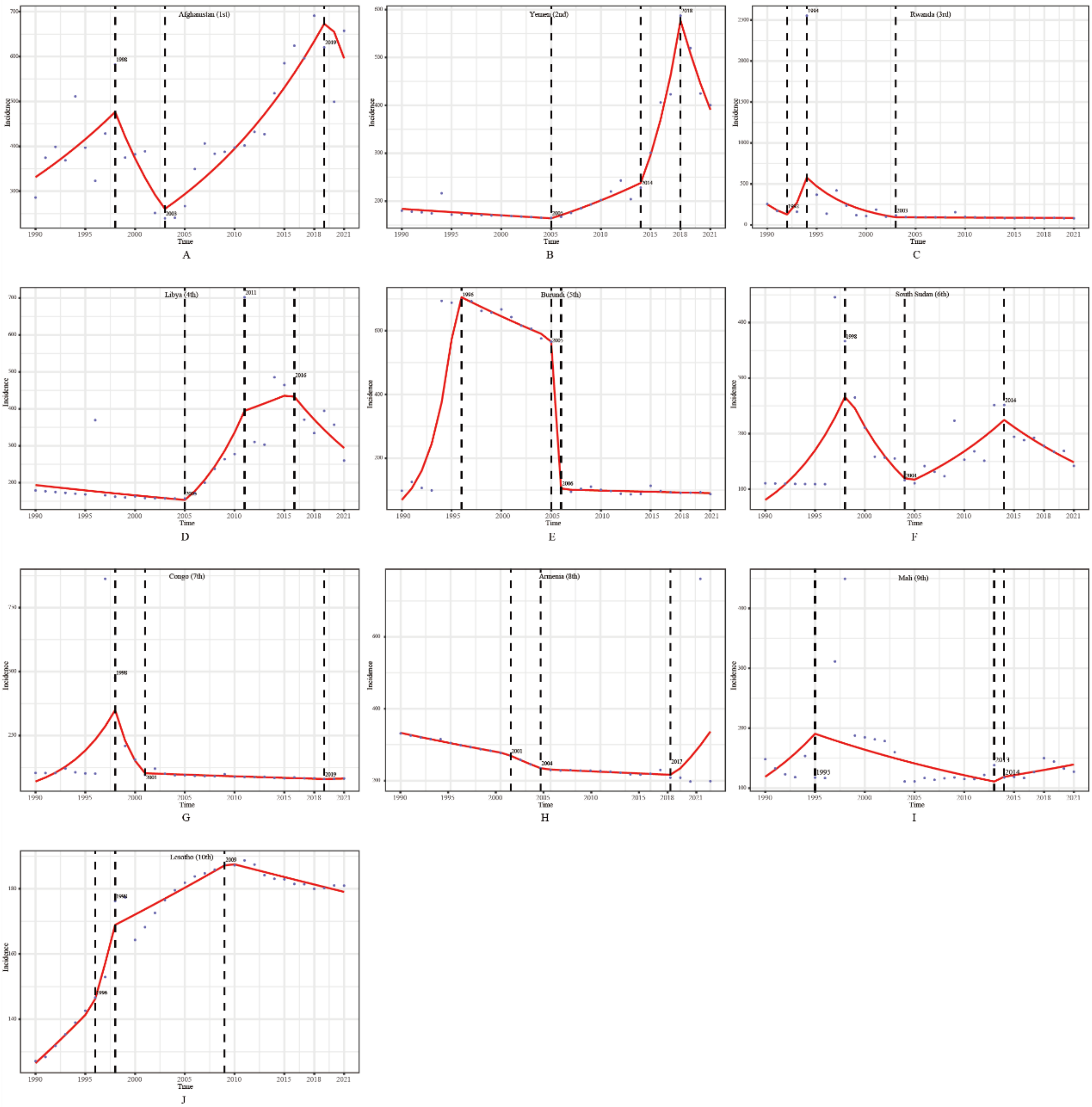
Afghanistan (1st)
Yemen (2nd)
Rwanda (3rd)
Libya (4th)
Burundi (5th)
South Sudan (6th)
Congo (7th)
Armenia (8th)
Mali (9th)
Lesotho (10th)
Incidence
Time
A
B
C
D
E
F
G
H
I
J

Figure S2:

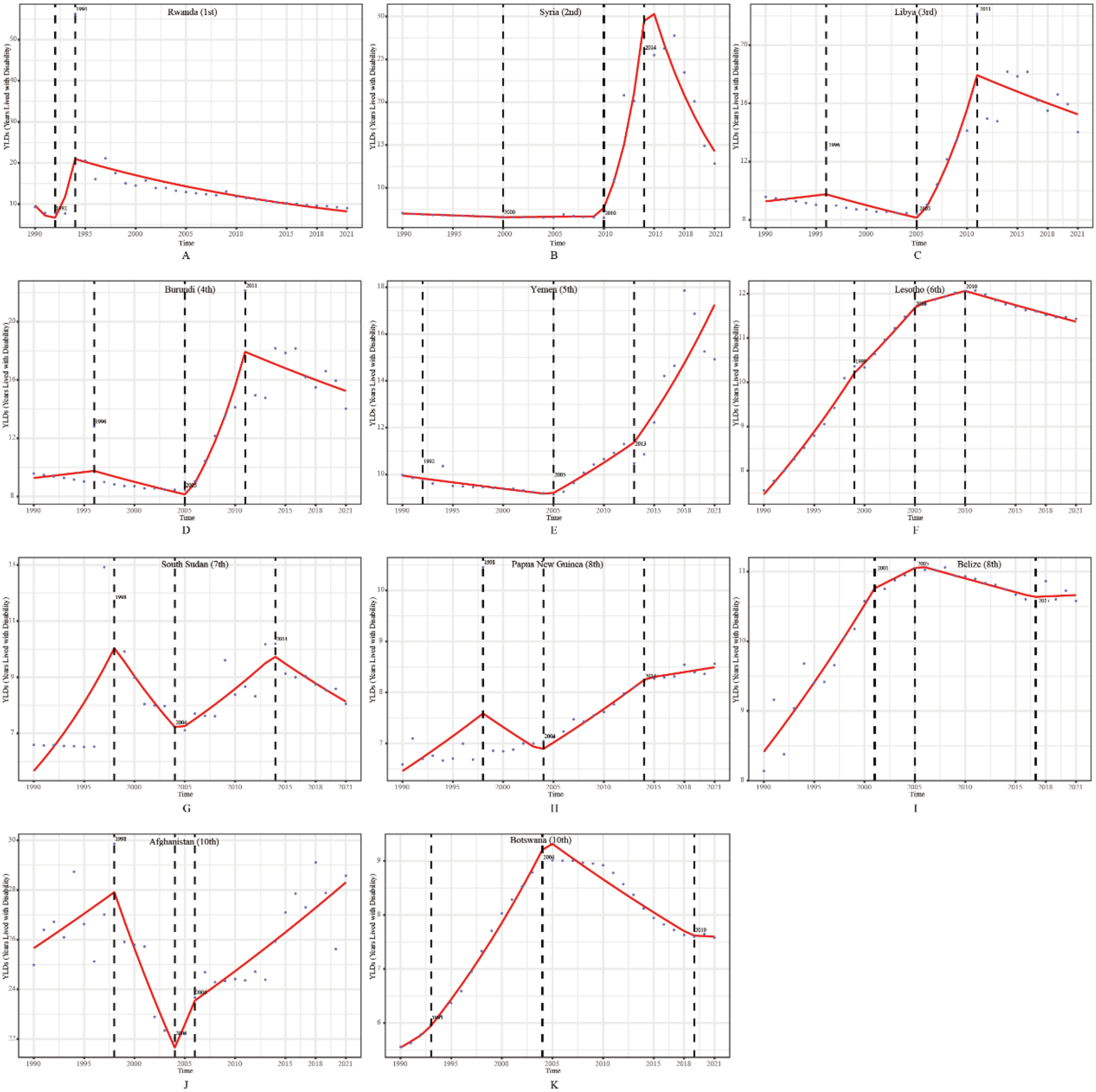

Rwanda (1st)
Syria (2nd)
Libya (3rd)
Burundi (4th)
Yemen (5th)
Lesotho (6th)
South Sudan (7th)
Papua New Guinea (8th)
Belize (8th)
Afghanistan (10th)
Botswana (10th)
YLDs (Years Lived with Disability)
Time
A
B
C
D
E
F
G
H
I
J
K

Figure S3:

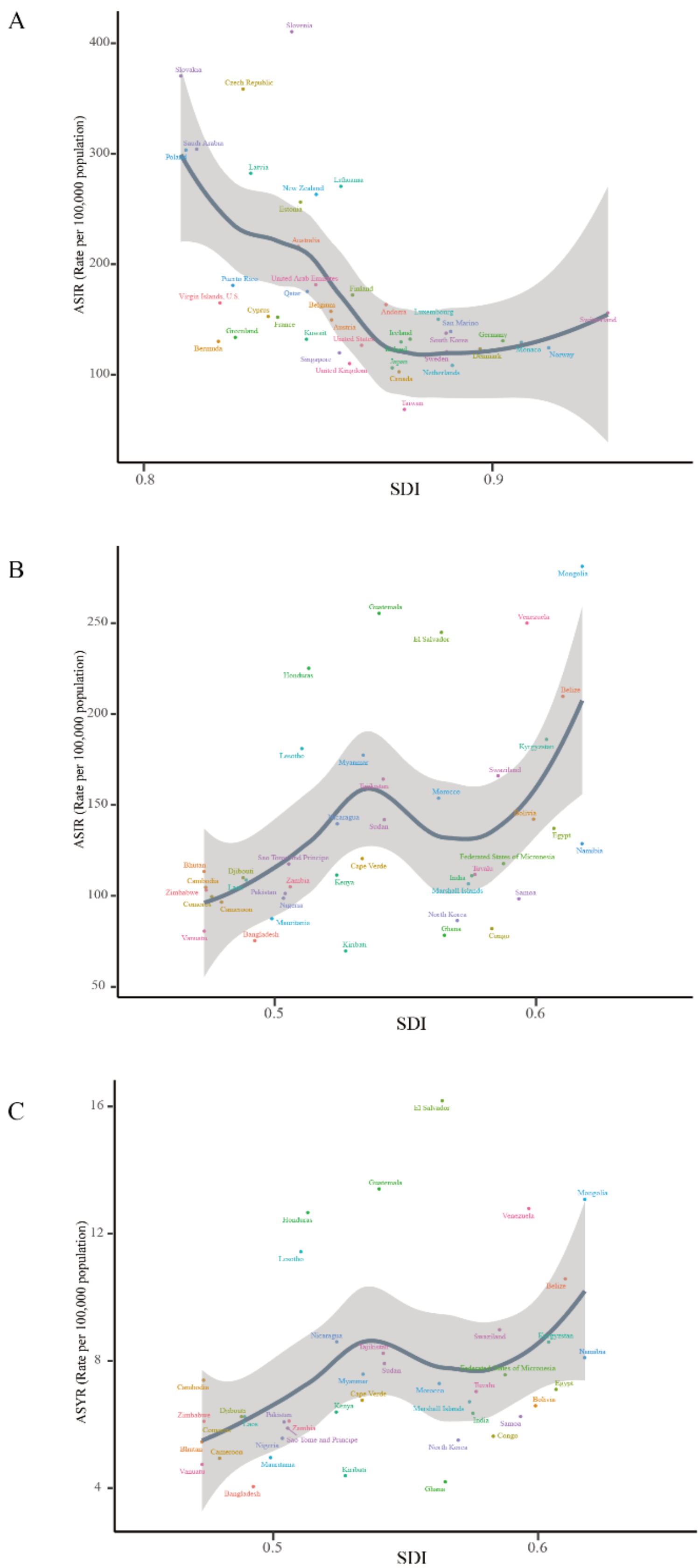
A
ASIR (Rate per 100,000 population)
400
300
200
100
0.8
0.9
SDI
B
ASIR (Rate per 100,000 population)
250
200
150
100
50
0.5
0.6
SDI
C
ASYR (Rate per 100,000 population)
16
12
8
4
0.5
0.6
SDI

Table S1. Level two/three cause proportion of ASIR of injury-associated internal hemorrhage in abdomen and pelvis (IAIHAP) in global and 5 SDI quintiles regions, 2021

Level two cause	Level three cause	global (%)		High SDI(%)		High-middle SDI(%)		Middle SDI(%)		Low-middle SDI(%)		Low SDI(%)	
Transport injuries	Road injuries	13.08	12.48	19.22	17.75	13.29	12.64	13.59	13.11	11.80	11.37	8.31	7.99
	Other transport injuries		0.60		1.47		0.66		0.48		0.43		0.32
Unintentional injuries	Falls	64.57	26.15	71.39	32.65	71.00	30.31	66.48	28.28	64.61	24.28	48.45	14.76
	Drowning		0.09		0.06		0.09		0.11		0.10		0.06
	Fire, heat, and hot substances		0.93		0.76		0.93		1.02		0.82		1.01
	Poisonings		0.09		0.23		0.13		0.08		0.05		0.04
	Exposure to mechanical forces		18.77		22.86		24.67		19.29		15.11		13.24
	Adverse effects of medical treatment		0.00		0.00		0.00		0.00		0.00		0.00
	Animal contact		3.97		2.69		2.20		3.10		6.66		5.67
	Foreign body		4.30		2.97		3.90		4.52		4.01		4.76
	Environmental heat and cold exposure		0.79		0.36		0.98		0.91		0.86		0.65
	Exposure to forces of nature		0.25		0.05		0.06		0.26		0.27		0.52
Self-harm and interpersonal violence	Self-harm	22.35	0.43	9.38	0.73	15.71	0.42	19.94	0.35	23.59	0.40	43.23	0.26
	Interpersonal violence		16.35		8.32		14.94		17.55		19.43		20.86
	Police conflict and executions		0.97		0.31		0.12		0.99		1.70		1.08
	Conflict and terrorism		4.60		0.02		0.22		1.04		2.06		21.03

ASIR, age-standardized-incidence-rate; SDI, sociodemographic index

Table S2. Level two/three cause proportion of ASYR of injury-associated internal hemorrhage in abdomen and pelvis (IAIHAP) in global and 5 SDI quintiles regions, 2021

Level two cause	Level three cause	global (%)		High SDI(%)		High-middle SDI(%)		Middle SDI(%)		Low-middle SDI(%)		Low SDI(%)	
Transport injuries	Road injuries	22.13	20.66	31.20	28.43	21.84	20.46	19.94	19.05	17.10	16.26	12.51	11.89
	Other transport injuries		1.47		2.77		1.39		0.88		0.84		0.63
Unintentional injuries	Falls	53.59	27.73	56.07	28.80	57.99	30.54	53.29	29.21	52.94	26.35	41.48	17.42
	Drowning		0.08		0.04		0.08		0.11		0.10		0.07
	Fire, heat, and hot substances		0.78		1.11		0.80		0.58		0.47		0.60
	Poisonings		0.11		0.17		0.14		0.09		0.06		0.06
	Exposure to mechanical forces		12.19		13.62		16.12		11.91		9.50		8.66
	Adverse effects of medical treatment		0.00		0.00		0.00		0.00		0.00		0.00
	Animal contact		3.58		3.93		2.35		2.48		5.51		5.25
	Foreign body		2.68		2.43		2.45		2.67		2.49		3.29
	Environmental heat and cold exposure		0.65		0.23		0.86		0.80		0.83		0.67
	Exposure to forces of nature		0.46		0.05		0.08		0.67		0.92		0.95
Self-harm and interpersonal violence	Self-harm	24.28	1.33	12.73	2.14	20.16	1.12	26.77	0.89	29.96	1.01	46.01	0.77
	Interpersonal violence		18.89		10.13		18.25		21.93		25.29		27.76
	Police conflict and executions		0.63		0.34		0.19		0.89		0.93		0.50
	Conflict and terrorism		3.43		0.11		0.60		3.06		2.73		16.98

ASYR,age-standardized-YLDs-rate; SDI, sociodemographic index; YLDs, years lived with disability.

Table S3. Cause proportion of ASIR of injury-associated internal hemorrhage in abdomen and pelvis (IAIHAP) in GBD region, 2021

	Unintentional injuries (%)	Transport injuries (%)	Self-harm and interpersonal violence (%)
Andean Latin America	81.49	10.18	8.32
Asia Pacific	74.76	16.70	8.54
Australasia	85.68	9.91	4.41
Caribbean	74.45	8.09	17.45
Central Asia	79.00	7.40	13.60
Central Europe	87.11	5.39	7.50
Central Latin America	67.11	9.56	23.33
Central Sub-Saharan Africa	52.36	19.74	27.89
East Asia	62.98	19.65	17.37
Eastern Europe	72.77	8.36	18.86
Eastern Sub-Saharan Africa	51.18	7.08	41.74
North Africa and Middle East	52.18	14.26	33.56
North America	55.88	31.92	12.20
Oceania	39.63	13.07	47.29
South Asia	68.79	9.80	21.41
Southeast Asia	64.55	11.90	23.55
Southern Latin America	73.90	16.56	9.54
Southern Sub-Saharan Africa	35.99	15.65	48.36
Tropical Latin America	63.73	11.43	24.84
Western Europe	77.81	15.43	6.76
Western Sub-Saharan Africa	55.43	8.54	36.03

ASIR, age-standardized-incidence-rate; SDI, sociodemographic index

Table S4. Cause proportion of ASYR of injury-associated internal hemorrhage in abdomen and pelvis (IAIHAP) in GBD region, 2021

	Unintentional injuries (%)	Transport injuries (%)	Self-harm and interpersonal violence (%)
Andean Latin America	68.10	16.78	15.12
Asia Pacific	60.47	27.90	11.63
Australasia	73.94	18.97	7.09
Caribbean	64.93	12.02	23.06
Central Asia	65.12	12.30	22.58
Central Europe	78.65	9.64	11.71
Central Latin America	51.20	14.18	34.62
Central Sub-Saharan Africa	39.05	26.69	34.26
East Asia	51.91	27.67	20.41
Eastern Europe	59.34	12.82	27.84
Eastern Sub-Saharan Africa	37.71	10.03	52.25
North Africa and Middle East	43.22	22.01	34.78
North America	41.42	43.42	15.16
Oceania	30.72	16.57	52.72
South Asia	59.09	14.17	26.73
Southeast Asia	53.51	17.62	28.87
Southern Latin America	57.94	28.01	14.05
Southern Sub-Saharan Africa	22.44	19.42	58.14
Tropical Latin America	50.20	16.40	33.40
Western Europe	63.60	27.05	9.35
Western Sub-Saharan Africa	43.61	12.35	44.03

ASYR,age-standardized-YLDs-rate; SDI, sociodemographic index; YLDs, years lived with disability.

Table S5. Cause proportion of top 10 countries/regions with highest AAPC of ASIR, non-peaceful period

	Unintentional injuries (%)	Transport injuries (%)	Self-harm and interpersonal violence (%)
Afghanistan (1st)	24.65	6.22	69.13
Yemen (2nd)	22.74	10.81	66.45
Rwanda (3rd)	9.91	2.88	87.21
Libya (4th)	20.78	7.69	71.53
Burundi (5th)	11.64	2.19	86.18
South Sudan (6th)	37.69	4.69	57.62
Congo (7th)	21.10	7.95	70.95
Armenia (8th)	64.63	4.21	31.16
Mali (9th)	51.71	6.66	41.64
Lesotho (10th)	38.37	13.73	47.90

AAPC, average annual percent change; ASIR, age-standardized-incidence-rate; SDI, sociodemographic index

Table S6. Cause proportion of top 10 countries/regions with highest AAPC of ASIR, the whole time			
	Unintentional injuries (%)	Transport injuries (%)	Self-harm and interpersonal violence (%)
Afghanistan (1st)	25.64	6.47	67.89
Yemen (2nd)	40.17	19.88	39.96
Rwanda (3rd)	28.14	7.10	64.76
Libya (4th)	37.97	14.72	47.32
Burundi (5th)	22.37	4.03	73.60
South Sudan (6th)	39.32	4.76	55.91
Congo (7th)	39.65	14.17	46.18
Armenia (8th)	76.73	4.71	18.55
Mali (9th)	44.17	5.35	50.48
Lesotho (10th)	34.77	13.76	51.47

AAPC, average annual percent change; ASIR, age-standardized-incidence-rate; SDI, sociodemographic index

Table S7. The correlation between ASIR/ASYR and SDI in global and 5 SDI quintiles regions in 2021

Location	ASIR and SDI		ASYR and SDI	
	Spearman's rho coefficient	P.Value	Spearman's rho coefficient	P.Value
Global	0.35	＜0.01	0.50	＜0.01
High SDI	-0.64	＜0.01	-0.20	0.22
High-middle SDI	0.22	0.14	0.25	0.09
Middle SDI	0.23	0.14	0.11	0.51
Low-middle SDI	0.46	＜0.01	0.52	＜0.01
Low SDI	-0.27	0.12	-0.09	0.61

ASIR, age-standardized-incidence-rate; ASYR,age-standardized-YLDs-rate; SDI, sociodemographic index; YLDs, years lived with disability.
