# Supplementary material for: Global, regional, and national burden of injury associated internal hemorrhage in the abdomen and pelvis from 1990-2021
Source: Commun Med (Lond). 2026 Apr 13;6:431. doi: 10.1038/s43856-026-01560-3 (PMC13444077; doi:10.1038/s43856-026-01560-3)
Supplement: Supplementary file 3 [file 43856_2026_1560_MOESM3_ESM.docx]

**Description of Additional Supplementary Files**

Supplementary Data 1: Incidence, prevalence, and years-lived-withdisability of injury-associated internal hemorrhage in abdomen and pelvis in 2021 and their average annual percentage changes from 1990 to 2021 at the global and regional levels

Supplementary Data 2: Incidence, prevalence, and years-lived-withdisability of injury-associated internal hemorrhage in abdomen and pelvis in 2021 and their average annual percentage changes from 1990 to 2021 at the national levels

Supplementary Data 3: List of International Classification of Diseases (ICD) codes mapped to injury causes in this study

Supplementary Data 4: GBD geographical hierarchy

Supplementary Data 5: SDI values and corresponding levels for each location in
2021

Supplementary Data 6: Incidence, prevalence, and years-lived-withdisability of injury-associated internal hemorrhage in abdomen and pelvis in 1990 at the global and regional levels

Supplementary Data 7: The AAPC of ASIR before and during COVID-19.

Supplementary Data 8: Data source for Figure 1.

Supplementary Data 9: Data source for Figure 2.

Supplementary Data 10: Data source for Figure 3.
